# Bacterial Biogeography across the Amazon River-Ocean Continuum

**DOI:** 10.3389/fmicb.2017.00882

**Published:** 2017-05-23

**Authors:** Mary Doherty, Patricia L. Yager, Mary Ann Moran, Victoria J. Coles, Caroline S. Fortunato, Alex V. Krusche, Patricia M. Medeiros, Jérôme P. Payet, Jeffrey E. Richey, Brandon M. Satinsky, Henrique O. Sawakuchi, Nicholas D. Ward, Byron C. Crump

**Affiliations:** ^1^Horn Point Laboratory, University of Maryland Center for Environmental Science, CambridgeMD, United States; ^2^Department of Marine Sciences, University of Georgia, AthensGA, United States; ^3^Josephine Bay Paul Center, Marine Biological Laboratory, Woods HoleMA, United States; ^4^Center of Nuclear Energy in Agriculture, University of São PauloPiracicaba, Brazil; ^5^College of Earth, Ocean, and Atmospheric Sciences, Oregon State University, CorvallisOR, United States; ^6^School of Oceanography, University of Washington, SeattleWA, United States; ^7^Massachusetts Institute of Technology, CambridgeMA, United States; ^8^Marine Sciences Laboratory, Pacific Northwest National Laboratory, SequimWA, United States

**Keywords:** Amazon River, tropical Atlantic Ocean, river plume, microbial diversity, freshwater bacteria, marine bacteria, diatom-diazotroph assemblage, Columbia River

## Abstract

Spatial and temporal patterns in microbial biodiversity across the Amazon river-ocean continuum were investigated along ∼675 km of the lower Amazon River mainstem, in the Tapajós River tributary, and in the plume and coastal ocean during low and high river discharge using amplicon sequencing of 16S rRNA genes in whole water and size-fractionated samples (0.2–2.0 μm and >2.0 μm). River communities varied among tributaries, but mainstem communities were spatially homogeneous and tracked seasonal changes in river discharge and co-varying factors. Co-occurrence network analysis identified strongly interconnected river assemblages during high (May) and low (December) discharge periods, and weakly interconnected transitional assemblages in September, suggesting that this system supports two seasonal microbial communities linked to river discharge. In contrast, plume communities showed little seasonal differences and instead varied spatially tracking salinity. However, salinity explained only a small fraction of community variability, and plume communities in blooms of diatom-diazotroph assemblages were strikingly different than those in other high salinity plume samples. This suggests that while salinity physically structures plumes through buoyancy and mixing, the composition of plume-specific communities is controlled by other factors including nutrients, phytoplankton community composition, and dissolved organic matter chemistry. Co-occurrence networks identified interconnected assemblages associated with the highly productive low salinity near-shore region, diatom-diazotroph blooms, and the plume edge region, and weakly interconnected assemblages in high salinity regions. This suggests that the plume supports a transitional community influenced by immigration of ocean bacteria from the plume edge, and by species sorting as these communities adapt to local environmental conditions. Few studies have explored patterns of microbial diversity in tropical rivers and coastal oceans. Comparison of Amazon continuum microbial communities to those from temperate and arctic systems suggest that river discharge and salinity are master variables structuring a range of environmental conditions that control bacterial communities across the river-ocean continuum.

## Introduction

The phylogenetic composition of microbial communities in freshwater and marine water predicts, to some degree, the genomic potential of communities in each of these environments (Zaneveld et al., 2011; Langille et al., 2013; Staley et al., 2014), and shapes the ecosystem services they provide in river and coastal ecosystems (Judd et al., 2006; They et al., 2013; Wear et al., 2014). Despite striking differences in the phylogenetic composition of freshwater and marine microbial communities (Crump et al., 1999; Logares et al., 2009), these communities are remarkably similar in ecological function and genomic character (Hobbie, 1988; Silveira et al., 2011; Fortunato and Crump, 2015). It is now clear that salinity influences the phylogenetic composition of microbial communities in aquatic systems (Troussellier et al., 2002; Lozupone and Knight, 2007; Herlemann et al., 2011; Fortunato et al., 2012; Campbell and Kirchman, 2013), but seasonal changes in other environmental factors are also thought to drive temporal patterns in community composition along freshwater-marine gradients (Crump and Hobbie, 2005; Kan et al., 2006; Fortunato et al., 2013). On seasonal time scales, microbial communities in surface oceans (Treusch et al., 2009; Eiler et al., 2011) and some lakes (Kent et al., 2004; Newton et al., 2006; Shade et al., 2007) track the same changes in light, water column stability, temperature, and nutrients that drive changes in phytoplankton communities. In contrast to surface oceans and lakes, the microbial community composition of flowing systems like rivers, estuaries, and river plumes is influenced by a broader range of seasonal factors including dissolved organic matter (DOM) quality and particulate organic matter concentration (Crump et al., 2009; Fortunato and Crump, 2011; Savio et al., 2015), and is more heavily influenced by dispersal and mixing of these microbial communities (Crump et al., 2004; Fortunato, 2012; Jackson et al., 2014; Read et al., 2015; Ruiz-Gonzalez et al., 2015).

The Amazon River is the largest river in the world, and accounts for 20% of the global freshwater discharge, transporting ∼36.1 Tg C y^-1^ of organic carbon to the Atlantic Ocean (Richey et al., 1990). Heterotrophic microbial respiration of terrestrially derived organic matter make these waters supersaturated with CO_2_, and contribute to large gas evasion fluxes of CO_2_ (Richey et al., 2002; Mayorga et al., 2005; Ward et al., 2015). The plume of the Amazon River extends for thousands of kilometers from the river mouth, and during peak seasonal discharge the plume can cover up to 1.3 × 10^6^ km^2^ of the western tropical Atlantic Ocean (Subramaniam et al., 2008). Observed and model drifters show that seasonally variable ocean currents carry this water far into the Caribbean and Gulf of Mexico as well as eastward to coastal Africa (Coles et al., 2013). As in the river, this large mass of water is associated with intense biological activity, delivering nutrients to support enhanced primary production (Demaster and Pope, 1996), including diatom blooms in lower salinity plume water, and large blooms of diatom-diazotroph assemblages (DDAs) at higher salinities (Carpenter et al., 1999; Subramaniam et al., 2008; Yeung et al., 2012). These blooms are associated with carbon export to the deeper ocean as they die off and sink to the bottom (Subramaniam et al., 2008; Yeung et al., 2012).

Bacterioplankton are major contributors to both the degradation of terrestrial organic carbon in the Amazon River (Tremblay and Benner, 2009; Ellis et al., 2012) and to nitrogen fixation (Foster et al., 2007), and carbon respiration in the Amazon plume with distinct ecological functions associated with particle-associated and free-living bacterial communities (Satinsky et al., 2014a). Despite their significance, the microbial communities extending along the gradient from the Amazon River to its plume in the western tropical North Atlantic and beyond have not been well characterized. Understanding the community composition and diversity of bacterioplankton along physiochemical and biological gradients will help identify ecological processes driving the production and transformation of organic matter in these regions, and will provide a test for the original River Continuum Concept, which hypothesized that microbial communities change along the aquatic continuum to adapt to the inefficiencies of upstream communities by forming new communities adapted to consume resources released from upstream environments (Vannote et al., 1980).

We used rRNA gene amplicon sequencing to characterize bacterial diversity in samples collected along the river-ocean continuum of the Amazon River and its plume in the western tropical North Atlantic Ocean. We collected samples in the lower Amazon River on three sampling trips in September and December 2010, and in May 2011, capturing the late declining (September), early rising (December), and maximum (May) river discharge periods (Lentz and Limeburner, 1995; Ward et al., 2013). Additionally, we sampled the Amazon plume along the gradient from low to high salinity water during peak discharge (May/June 2010) and during the decline of the seasonal discharge (September 2011). We also sampled a large DDA bloom in the plume in May/June 2010. Our results suggest that bacterial communities in the Amazon River are shaped by environmental factors controlled by seasonality in river discharge, while communities in the Amazon plume are insensitive to seasons and instead are loosely structured by salinity, which provides a proxy for the evolving inorganic and organic constituents of the plume.

## Materials and Methods

### Sample Collection

**FIGURE 1:**
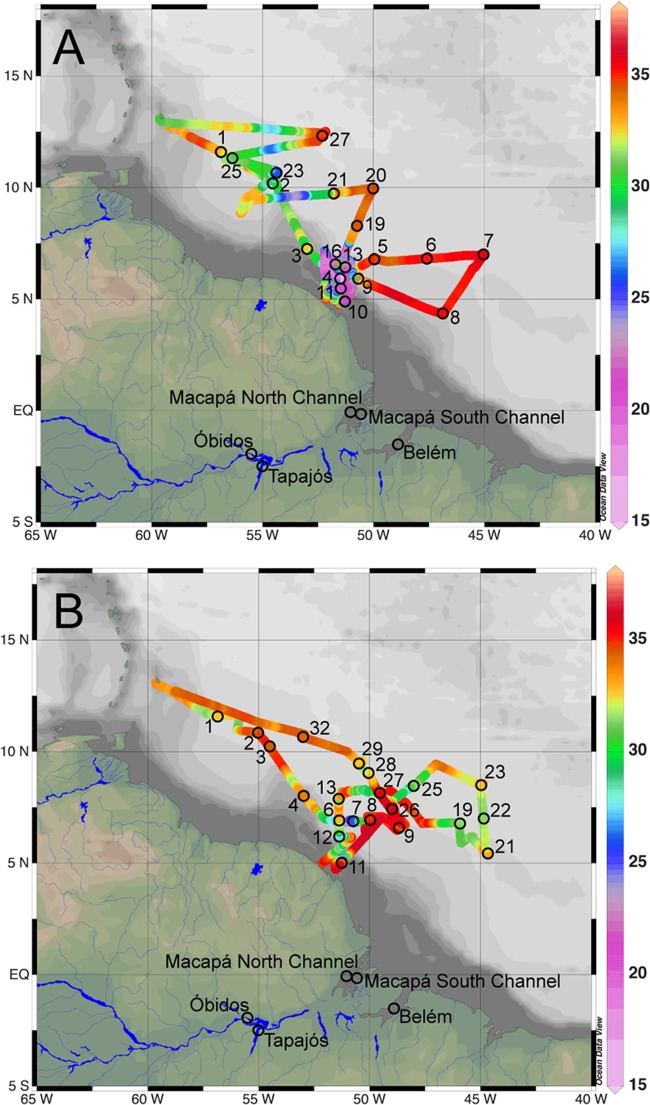
**Map of sampling stations from cruises in the Amazon River plume aboard the R/V Knorr in May/June 2010** **(A)**, and the R/V Melville in September 2011 **(B)**. Amazon River sampling stations from September 2010, December 2010, and May 2011 are indicated on both maps. Surface water salinity along cruise tracks are indicated with colors.

Amazon River samples were collected on three cruises (September 2010, December 2010, and May 2011) from five stations: an upriver station (Óbidos), a major lower Amazon tributary river station (Tapajós), and near the mouth (Macapá North, Macapá South, and Belém) using methods described in Ward et al. (2015) (**Figure 1** and Supplemental Table S1). In brief, water samples were collected from the surface and 50% depth (i.e., half the water column depth) at three cross-channel stations for each sampling site using a Shurflo submersible pump with a 297 μm pore-size mesh screen. Here we defined the Amazon River mouth as the last two well-constrained channels near the city of Macapá, which is ∼150 km from the highly channelized actual river mouth. The Amazon River also mixes with the Tocantins River, south of the Marajó Island, discharging to the ocean near the city of Belém. Although this channel is not typically considered to be part of the Amazon River, its discharge travels north and is integrated with the plume. Roughly 50% of the total river discharge to the plume occurs through Macapá South, 30% through Macapá North, and 20% through Belém (Ward et al., 2015).

Water samples from the Amazon River plume were collected from the R/V Knorr on May 23 to June 21, 2010, and from the R/V Melville on September 5 to October 6, 2011 (**Figure 1** and Supplemental Table S1) using 20 L Niskin bottles or a submersible pump. Cruise tracks were selected to sample the Amazon River plume, including associated DDA blooms, and to capture the extent of the plume’s influence in both depth and distance from the river mouth.

Three types of DNA samples were collected at each station. Cells in screened (river, 297 μm mesh) and un-screened (plume) water samples were collected on 0.2 μm Sterivex filters (Millipore, Billerica, MA, United States), and flooded with RNAlater preservative (Applied Biosystems, Austin, TX, United States). Cells were also partitioned into two size fractions by sequential filtration through 2.0 μm pore-size, 142 mm diameter polycarbonate (PCTE) membrane filters (Sterlitech Corporation, Kent, WA, United States) and 0.22 μm pore-size, 142 mm diameter Supor membrane filters (Pall, Port Washington, NY, United States). These filters were immediately submerged in RNAlater in sterile 50 mL tubes. Filtration and stabilization of all samples was completed within 30 min of water collection. Following RNAlater addition, all filters were incubated at room temperature overnight, frozen, dry-shipped in liquid nitrogen dewars, and stored at -80I°C until extraction. River samples were initially stored at -20°C before shipping to the United States.

### Environmental Measurements

River discharge, dissolved organic carbon (DOC) and total dissolved nitrogen (TDN) were determined as described in Ward et al. (2015). River temperature and pH were measured using a Thermo Orion 290A Plus meter with the probe immersed in an overflowing graduated cylinder. River conductivity and dissolved oxygen were measured using an Amber Science 2052 m and a YSI 55 m, respectively, with the probes immersed in the same graduated cylinder. River ion concentrations (NH_4_^+^, NO_3_^-^, NO_2_^-^, PO_4_^-^, and Cl^-^) were determined by flow injection analysis using a Foss-Tecator FiaStar 5000A FIA Analyzer with samples that were filtered through Whatman cellulose acetate filters (0.45 μm pore-size) into acid-washed 60 mL high-density polyethylene bottles, preserved with thymol, and frozen at -20°C until analysis. Major ions (Si, SO^4+^, Na^+^, K^+^, Mg^+^, and Ca^+^) were measured in the same samples using a Horiba-Jobyn Ivon UltimaPro inductively coupled plasma-optical emission spectroscope. Dissolved inorganic carbon (DIC) concentrations were measured on a Shimadzu total carbon analyzer (Model TOC-VCPH) using water samples filtered through 0.47 μm cellulose acetate membrane filters (Millipore) into acid-washed 60 mL HDPE bottles with no headspace to avoid degassing and preserved with thymol (100 mg/1000 ml of solution). DIC samples were analyzed for total organic carbon before and after acidification with 6 N HCl and sparging to remove gas; DIC was calculated as the difference between non-acidified and acidified/sparged samples. Finally, river dissolved CO_2_ concentrations were calculated based on DIC, pH, and temperature measurements.

Plume measurements of salinity, temperature, fluorescence, and nutrients (SiO_3_, NO_3_+NO_2_, and PO_4_) were described in Coles et al. (2013), Goes et al. (2014), and Weber et al. (2016). Plume measurements of DOC, chlorophyll a, bacterial abundance, and bacterial production were described in Medeiros et al. (2015) and Seidel et al. (2015). Plume measurements of particulate organic carbon and nitrogen were made by continuous-flow isotope-ratio mass spectrometry (CF-IRMS) using a Micromass Optima interfaced to a CE NC2500 elemental analyzer as described in Loick-Wilde et al. (2016).

Student’s *t*-tests were performed using Microsoft Excel to determine statistically significant differences between biogeochemical parameters across the study boundaries within a 95% confidence interval.

### DNA Extraction

DNA was extracted using methods adapted from Zhou et al. (1996) and Crump et al. (2003) with the following modifications. For Sterivex filters, RNAlater preservative was pushed out of the filter cartridge with a sterile syringe, and the filter was triple-rinsed with either sterile water (river) or sterile 0.1% phosphate-buffered saline (PBS; plume) to remove residual RNAlater. The filter was then removed from the casing by cracking the housing with pliers, sliced on a sterile cutting board, placed in a 2 ml tube, and submerged with ∼1 ml of DNA extraction buffer (DEB: 0.1 M Tris-HCl (pH 8), 0.1 M Na-EDTA (pH 8), 0.1 M Na_2_H_2_PO_4_ (pH 8), 1.5 M NaCl, 5% CTAB) (Zhou et al., 1996). Each 142 mm 0.22 μm pore-size Supor filter was removed from RNAlater, placed in a sterile plastic Whirlpak bag, flash frozen at -80°C, and shattered. Each 142 mm 2.0 μm pore-size PCTE filter was removed from RNAlater, folded, and sliced on a sterile cutting board. Pieces of filters were triple-rinsed with a sterile solution (PBS or H_2_O) in 50 mL conical tubes. Dislodged cells in RNAlater and rinse solutions were collected on Sterivex filters, triple-rinsed with sterile solution (PBS or H_2_O), processed as above, added to the 142 mm filter pieces, and submerged in 7–9 mL DEB. *Thermus thermophilus* strain HB8 (ATCC) genomic DNA was added to each sample once the filter was placed in DEB to serve as an internal standard to account for variable extraction efficiency (Satinsky et al., 2013). *T. thermophilus* DNA was added at 0.01% by mass of the expected DNA recovery from each sample, which was calculated from volume filtered assuming 10^6^ bacterial cells/L and 1 Mb average genome size (Biers et al., 2009). DNA was then extracted according to Crump et al. (2003) with modifications made for the larger volumes used with the 142 mm filters.

### Amplicon Pyrosequencing and Data Analyses

DNA from this study and from six arctic rivers (previously analyzed with a different method; Crump et al., 2009) was PCR-amplified using primers for bacterial 16S ribosomal RNA genes in three or four replicate 20 μl reactions. These bacteria-specific primers, targeting the V1–V2 regions, were 27F with 454B FLX linker (GCCTTGCCAGCCCGCTCAG *TC* AGRGTTTGATYMTGGCTCAG) and 338R with 454A linker and unique 8 base pair barcode denoted as ‘N’ (GCCTCCCTCGCGCCATCAG NNNNNNN *CA* TGCWGCCWCCCGTAGGWGT) (Modified from Hamady et al., 2008). Replicate amplicons were combined, quantified, pooled in normalized masses, purified either with MoBio Ultraclean PCR Cleanup Kits (MoBio Laboratories, Solana Beach, CA, United States), or S.N.A.P. UV-Free Gel Purification Kits (Invitrogen, Carlsbad, CA, United States) using 0.8% agarose gels, and pyrosequenced on a Roche-454 FLX Pyrosequencer at Engencore at the University of South Carolina using titanium chemistry^1^. DNA sequence data was deposited at NCBI^2^ under BioSample accessions SAMN06102005-SAMN06102159.

Sequence data was quality controlled and analyzed on the Data Intensive Academic Grid (DIAG) shared computational cloud at the University of Maryland School of Medicine Institute for Genome Sciences (IGS) following Fortunato et al. (2013) and using AmpliconNoise v1.24 (Quince et al., 2011), MacQiime v1.6.0 (Caporaso et al., 2010), Mothur (Schloss et al., 2009), PRIMER v6 (PRIMER-E Ltd, Plymouth, United Kingdom), and R (v2.14.0). Quality control used the AmpliconNoise pipeline with recommended procedures for Titanium sequencing chemistry. Maximum sequence length was set to 250 bp (Parse.pl), and chimera were identified and removed (PerseusD). Sequences were clustered into operational taxonomic units (OTUs) with the uclust option in QIIME (pick_otus.py) based on 97% sequence identity, and taxonomy was determined with the RDP Classifier retrained to use the Silva 111 database (Pruesse et al., 2007), and to a custom freshwater bacterial database (Newton et al., 2011) modified by Katherine McMahon (personal communication). OTUs identified as internal standard (*T. thermophilus*), chloroplast, mitochondria, Archaea, and unclassified were removed and the remaining OTUs were rarified to 887 sequences per sample. Alpha-diversity was estimated using Catchall (Bunge, 2011), which computes maximum likelihood estimates of diversity based on a suite of parametric and non-parametric models. Beta-diversity was estimated using Bray–Curtis similarity (Clarke, 1993) and weighted and unweighted UniFrac distance analyses (Lozupone et al., 2006). Similarity matrices were visualized using multiple dimensional scaling (MDS) diagrams, and differences between *a priori* groups of samples were tested with Analysis of Similarity statistics (ANOSIM; Clarke, 1993). OTUs that characterize each sample group were identified with Indicator Species Analysis using labdsv (Roberts, 2016) and indval packages (Dufrene and Legendre, 1997) in R.

To determine environmental factors that explain community variability, environmental data (Supplemental Tables S2, S3) were converted to *Z*-scores and analyzed using BV-STEP and BIO-ENV (Clarke and Ainsworth, 1993) (ρ > 0.95, Δρ < 0.001, 24 starting factors for river and 11 starting factors for plume). These analyses calculate Spearman rank correlation coefficients (ρ) to determine the degree of association between OTU similarity matrices and tables of environmental factors. The percent of community variability explained by factors identified by BV-STEP was determined with canonical correspondence analysis (CCA) (Ter Braak, 1986), or with redundancy analysis (RDA) when community data varied linearly along environmental gradients (Legendre and Anderson, 1999). Pairwise correlation coefficients were calculated for the environmental data to ensure that highly correlated variables (>0.9, <-0.9) were not included in the analysis.

Inferred bacterial associations (co-occurrence and mutual exclusion) within plume and river samples were computed using the CoNet (v1.1.1.beta) plugin within Cytoscape (v3.4.0)^3^ as previously described (Faust et al., 2012; Faust and Raes, 2016). Two separate association networks were constructed using rarefied OTU tables for plume and river. For each network, co-occurrence and mutual exclusion associations were identified using an ensemble of correlation (Spearman and Pearson coefficients) and distance (Bray–Curtis and Kullback–Leibler dissimilarity measures) metrics. For each association metric and each edge, 100 renormalized permutation and bootstrap scores were generated following the ReBoot procedure developed by Faust et al. (2012). The measure-specific *p*-values from multiple association metrics were merged using the Simes method (Sarkar and Chang, 1997) and false-discovery rate correction was performed using Benjamini–Hochberg multiple testing correction (Benjamini and Hochberg, 1995). Only 1000 top- and 1000 bottom-ranking edges from each association measure were kept in the network analysis, and only edges supported by at least two of the four association metrics were retained in the final network inference of associations among taxa.

## Results

### River and Plume Physicochemical Conditions

Amazon river biogeochemistry varied by season and by sampling site (Supplemental Table S2). River discharge was in the late declining phase in September 2010, and in the very early rising stage in December, reaching maximal discharge in May 2011. Temperature, conductivity, pH, and oxygen saturation were negatively related to discharge at most sites. During each sampling period temperature was similar at all sites, but conductivity, DIC, chlorine, sulfate, sodium, potassium, and magnesium were higher in the mainstem (Óbidos and Macapá) compared to the Tapajós River. pH was similar at Óbidos and Macapá, and was different (higher or lower) at Belém and in the Tapajós River on most sampling dates. Dissolved CO_2_ was highest in the mainstem, decreasing slightly from Óbidos to Macapá. Dissolved oxygen was lower in the turbid mainstem, with lowest values at Óbidos increasing toward the mouth at Macapá and Belém. The highest values were observed in the Tapajós where oxygen saturation approached 100% in September. DIN, nitrate, and ammonium concentrations were highly variable and followed no clear trends, but nitrite was positively related to river discharge. DOC and DON peaked in May in the Tapajós River and at Óbidos, but were otherwise similar across all stations and sampling dates.

Amazon River plume biogeochemistry varied as a function of river dilution, reflected in salinity, and was similar between the high river discharge in May 2010 and low discharge in September 2011 (Supplemental Table S3). Temperature varied by only 2.1°C across all samples. Oxygen saturation was near 100% or greater in all plume samples and dissolved oxygen concentrations were negatively related to salinity as were DOC, POC, PN, phosphate, silica, bacterial abundance, and bacterial production. However, nitrate+nitrite and phosphate concentrations did not track salinity and were notably lower in May than September (*t*-test, *p* < 0.05). Chlorophyll *a*, fluorescence, POC, and PN peaked in low salinity and DDA samples.

### Alpha- and Beta-Diversity

**FIGURE 2:**
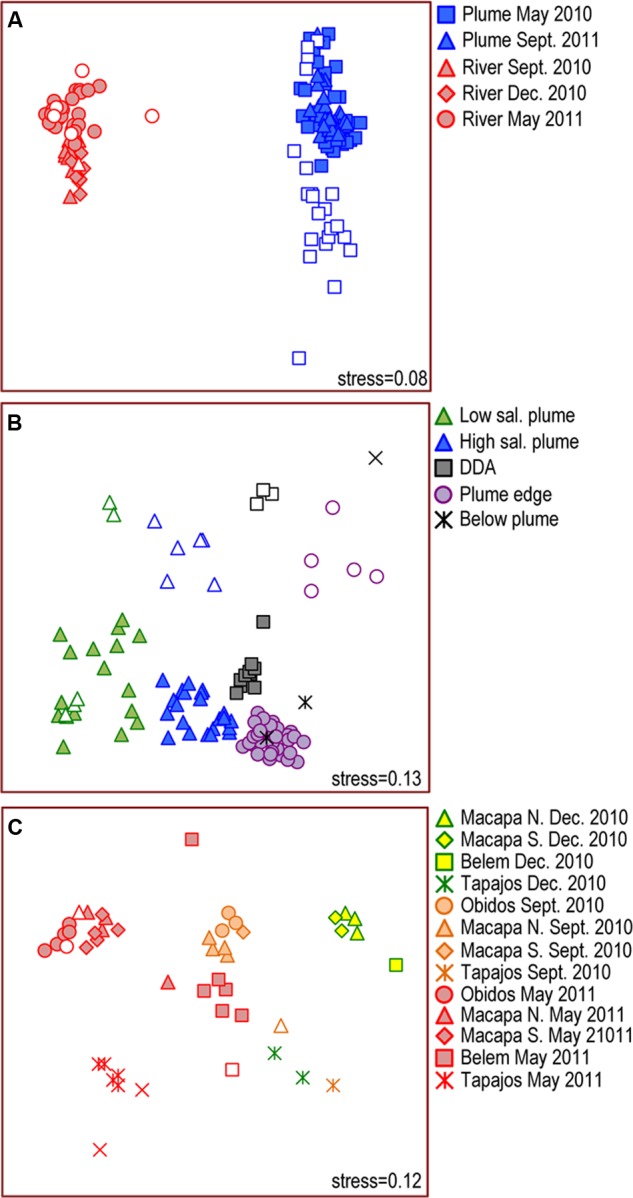
**Multidimensional Scaling (MDS) diagrams showing Bray–Curtis similarity among bacterial communities in** **(A)** all samples collected in the Amazon River and Plume in 2010 and 2011, **(B)** Amazon plume samples, and **(C)** Amazon River samples. Closed symbols represent unfractionated samples and free-living bacteria (0.2–2.0 μm). Open symbols represent particle-attached bacteria (>2.0 μm).

The greatest variability in bacterial community composition was between river and plume (**Figure 2A**), and, among plume samples, was between size fraction (>2.0 μm and 0.2–2.0 μm) (ANOSIM, *R* = 0.66, *P* < 0.001) (**Figure 2B**), with the exception of low salinity station 10 in 2010 (salinity 22.6) where the >2.0 μm fraction was similar to the 0.2–2.0 μm fraction. Communities in unfractionated “whole” water samples grouped with the 0.2–2.0 μm fractions (**Figure 2A**). In contrast, there was no significant difference between the size fractions among river samples (ANOSIM, *R* = 0.10, *P* = 0.478; **Figure 2C**).

Plume communities in the 0.2–2.0 μm fraction clustered into four groups (ANOSIM, *R* = 0.82, *P* < 0.01) with overlapping salinity ranges: near-shore low salinity plume (salinity range: 20.9–29.5), off-shore high salinity plume (salinity range: 26.5–35.2), DDA blooms (salinity range: 31.9–32.5), and the plume edge (salinity range: 31.1–36.1, **Figure 2B** and Supplemental Figure S5). Communities in deep samples collected below the plume were most similar to communities at the plume edge. Particle-associated communities in the >2.0 μm size fraction also clustered into four groups (ANOSIM, *R* = 0.93, *P* < 0.001) matching those in the 0.2–2.0 μm fraction (**Figure 2B**). There was no clear seasonal change in plume communities except that the DDA assemblage was only observed in May/June 2010.

River communities grouped primarily by sampling date (**Figure 2C** and Supplemental Figure S5). Communities in mainstem river samples from Óbidos, Macapá North, and Macapá South grouped together strongly, but were different in composition on each sampling date (ANOSIM, *R* = 0.95, *P* < 0.001) (**Figure 2C**). Communities in the Tapajós River and at some Belém stations were distinct from the mainstem stations (ANOSIM, *R* = 0.40, *P* < 0.001), and also differed between dates (ANOSIM, *R* = 0.85, *P* < 0.001). We investigated cross-channel variability at mainstem stations by sampling the left, middle, and right side of the channel, and found no significant cross-channel variability at Óbidos and Macapá. At Belém, communities at the center and right stations were different than those on the left side of the channel in May 2011 (**Figure 2C**), likely due to mixing with Tocantins River communities.

Alpha-diversity (Catch-all) was significantly greater in the river than in the plume (Supplemental Figure S1), and ranged from 255 to 1160 OTUs (median = 697, mean = 706, and CV = 0.31). In comparison, plume alpha-diversity ranged from 85 to 596 OTUs (median 222, mean 232, and CV 0.40). The estimated number of OTUs in 83% (40/48) of the river samples was significantly greater than 81% (85/105) of the plume/ocean samples, based on a comparison of the overlap between the calculated confidence intervals in the two datasets. This pattern held regardless of sampling dates, size fractions, and the groups identified based on community composition.

### Comparison with Environmental Data

The best model explaining variability in river communities included eight environmental factors (**Table 1**) among which conductivity (ρ = 0.553) and pH (ρ = 0.566) were the highest ranked. CCA analysis showed that conductivity and pH explain 34.2% of the variability. For mainstem river communities (Macapá North, Macapá South, and Óbidos) the best model included six factors, with discharge (ρ = 0.906) and pH (ρ = 0.748) explaining 33.9% of variability. For tributary communities (Tapajós and Belém), the best model included 17 factors, with Cl^-^ (ρ = 0.710) and Si (ρ = 0.678) explaining 66.2% of variability. For Tapajós communities alone, the best model included three factors, with discharge (ρ = 0.785) and Si (ρ = 0.777) explaining 60.4% of variability.

**Table 1:** Spearman Rank coefficients (ρ) showing correlations between environmental data and bacterial community composition for all river samples, and samples grouped by location in the mainstem Amazon River (Macapá North, Macapá South, and Óbidos), tributaries (Tapajós and Belém), and the Tapajós tributary alone.

Environment	*N*	BV-STEP factors	ρ	BIO-ENV factors	ρ	Variability explained
All	46	Conductivity, dissolved oxygen, temperature, pH, DON, chlorine, iron, discharge	0.776	pH	0.566	23.4%^∗^
				Conductivity	0.553	
Mainstem	30	Dissolved oxygen, temperature, pH, DON, ammonia, discharge	0.910	Discharge	0.906	25.5%^∗^
				DO	0.768	
Tributaries	16	Conductivity, dissolved oxygen, temperature, pH, DOC, pCO2, Total N, DON, chlorine, nitrite, nitrate, sodium, magnesium, calcium, silicon, iron, discharge	0.686	Chlorine	0.710	64.9%^∗^
				Discharge	0.678	
Tapajós	9	Temperature, DOC, DIN	0.777	Discharge	0.785	61.7%^∗^
				Silicon	0.777	


*BV-STEP ρ values represent maximum values when all environmental variables were included. For BIO-ENV, the maximum number of variables was set to one to determine the correlation for each individual variable, and ρ values for the top two variables are shown. The environmental variables from BV-STEP results were used in canonical correspondence analysis (CCA) to determine the percent of the variability explained (^∗^*P* < 0.005).*

The best model explaining variability in plume communities included five environmental factors, but the correlation was not strong (ρ = 0.540; **Table 2**) likely because of the difference between the two size fractions. For the 0.2–2.0 μm size fraction and unfractionated samples the best model included four factors, with salinity (ρ = 0.749) and bacterial production (ρ = 0.790) explaining 13.6% of variability, based on CCA. For communities in the >2.0 μm size fraction the best model included two factors, with fluorescence (ρ = 0.494) and chlorophyll-*a* (ρ = 0.486) explaining 11.3% of the variability (**Table 2**). Variability in plume bacterial communities was better explained by environmental factors when major groupings of communities were analyzed separately (**Table 2**).

**Table 2:** Spearman rank coefficients (ρ) showing correlations between environmental data and bacterial community composition for all plume and ocean samples, samples grouped by size fraction (whole, 2.0 and 0.2 μm), and samples grouped by location for each size fraction [low salinity plume, high salinity plume, plume edge, and diatom-diazotroph assemblage (DDA)].

Environment	*N*	BV-STEP factors	ρ	BIO-ENV factors	ρ	Variability explained
All	92	Depth, salinity, oxygen, Si, BP	0.540	Salinity	0.481	5.3%
				BP	0.479	
Whole & 0.2—2.0 μm	72	Salinity, oxygen, fluorescence, BP	0.830	BP	0.790	13.6%
				Salinity	0.749	
Low salinity plume	13	Temperature, salinity, fluorescence, PO_4_, DOC	0.690	DOC	0.749	51.4%
				PO_4_	0.579	
High salinity plume	22	Depth, salinity, oxygen, chlorophyll, silicate, NO_3_+NO_2_, BP, DOC	0.713	BP	0.564	45.4%^∗^
				O_2_	0.555	
DDA	8	Fluorescence, silicate	0.442	BP	0.435	29.1%
				Bacterial count	0.435	
Plume edge	29	Depth, salinity, oxygen, Si, bacterial count	0.464	Salinity	0.470	10.8%
				Silicate	0.348	
>2.0 μm	20	Fluorescence, chlorophyll, NO_3_+NO_2_	0.508	Fluorescence	0.494	12.6%
				Chlorophyll	0.486	
High salinity plume	6	Temperature, salinity, bacterial count, DOC	0.603	PO_4_	0.746	63.1%
				Bacterial count	0.574	
DDA	4	Depth	0.500	Depth	0.500	7.7%
				Fluorescence	0.500	
Plume edge	5	PO_4_	0.960	PO_4_	0.960	67.2%
				Fluorescence	0.859	


*BV-STEP ρ values represent maximum values when all environmental variables were included. For BIO-ENV, the maximum number of variables was set to one to determine the correlation for each individual variable, and ρ values for the top two variables are shown. Canonical redundancy analyses (RDA) were performed with environmental variables identified in BV-STEP to obtain the percent variability explained (^∗^*P* < 0.005).*

### Taxonomic Composition and Indicator Taxa

**FIGURE 3:**
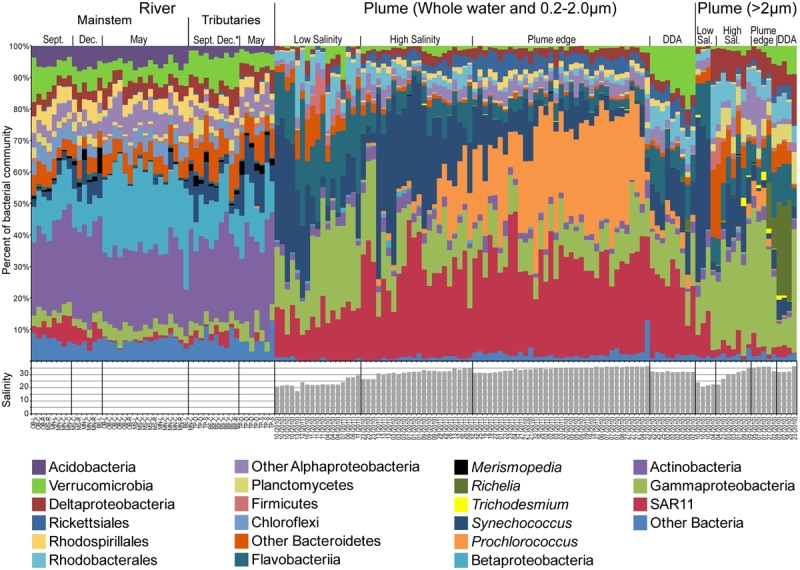
**Taxonomic diversity and salinity of Amazon River and Plume samples grouped by environment following **Figure 2**, and showing the most abundant taxonomic groups.** River samples are ordered by month in the mainstem river (Óbidos and Macapá), and tributary stations (Belém and Tapajós). Plume samples are ordered by environmental group for the 0.2–2.0 μm and whole water samples, followed by the >2.0 μm fraction samples.

In the mainstem Amazon River, Actinobacteria formed the highest proportion of sequences (average = 25.8%), and Betaproteobacteria shifted seasonally, peaking during high discharge in May (**Figure 3** and Supplemental Figure S2). Indicator taxa (*P <* 0.01) reflected this shift (Supplemental Table S4 and Figures S3, S4); December indicator taxa included a diverse group of Actinobacteria (acI-C and acIV-A), and May indicator taxa included a diverse group of Betaproteobacteria (*Limnohabitans* betI, *Polynucleobacter* betII, and *Methylophilales* betIV). Seasonal shifts were also observed for freshwater relatives of the Alphaproteobacteria SAR11, and Cyanobacteria (mainly *Synechococcus* and *Merismopedia*), both of which had highest proportions during low discharge (4.9 and 5.3%, respectively). Cyanobacteria peaked in abundance in tributaries in the Tapajós and the Belém center and right bank stations (average 12.7% low discharge, 12% high discharge) (**Figure 3**), and were among the indicator taxa for tributaries in September and December. In the mainstem river, indicator taxa accounted for large proportions of sequences in December (7%) and May (9%), but there were very few significant indicator taxa for September, representing only 1.9% of sequences (Supplemental Table S4).

Plume communities in the 0.2–2.0 μm and >2.0 μm size fractions varied along the salinity gradient, but did not closely track salinity (**Figure 3**). Freshwater taxa were almost entirely absent from plume samples, averaging 0.001% of communities in samples with salinity <30, with the exception of one Actinobacteria OTU that was most abundant in both freshwater and high salinity plume samples and therefore likely represents closely related freshwater and marine taxa. The low salinity plume community was dominated by SAR11, Flavobacteriia, Gammaproteobacteria, and *Synechococcus*, and some samples included Sphingobacteriia and Firmicutes. High salinity plume communities were dominated by *Synechococcus* and SAR11, and many of the dominant low salinity taxa were rare or absent. As sample salinity increased toward the plume edge, the proportion of *Synechococcus* declined and the proportion of *Prochlorococcus* increased (**Figure 3**). Indicator taxa reflected this taxonomic shift between the low salinity plume and the plume edge (Supplemental Table S4 and Figures S3, S4). Indicators for the low salinity plume (both size fractions) included a very abundant *Synechococcus*, and several Gammaproteobacteria, SAR11, and Bacteroidetes (Flavobacteriia and Sphingobacteriia). High salinity plume had few indicator taxa other than one very abundant *Synechococcus* in the 0.2–2.0 μm fraction and several Sphingobacteriia in the >2.0 μm size fraction. Plume edge indicator taxa were dominated by *Prochlorococcus* and Alphaproteobacteria (SAR11 and Rickettsiales) in the 0.2–2.0 μm fraction and an abundant Gammaproteobacteria in the >2.0 μm size fraction. In DDA communities, the proportion of Verrucomicrobia and Flavobacteriia was elevated and *Synechococcus* and *Prochlorococcus* were reduced (**Figure 3**). In the >2.0 μm fraction, DDA communities were very similar to high salinity communities, with the addition of the same Verrucomicrobia found in the 0.2–2.0 μm size fraction, and the Cyanobacteria *Richelia*, which is a diazotroph symbiont of DDA (Hilton et al., 2015). DDA bloom indicators included *Richelia* in the >2.0 μm size fraction and many Verrucomicrobia and Flavobacteriia in the 0.2–2.0 μm fraction.

**FIGURE 4:**
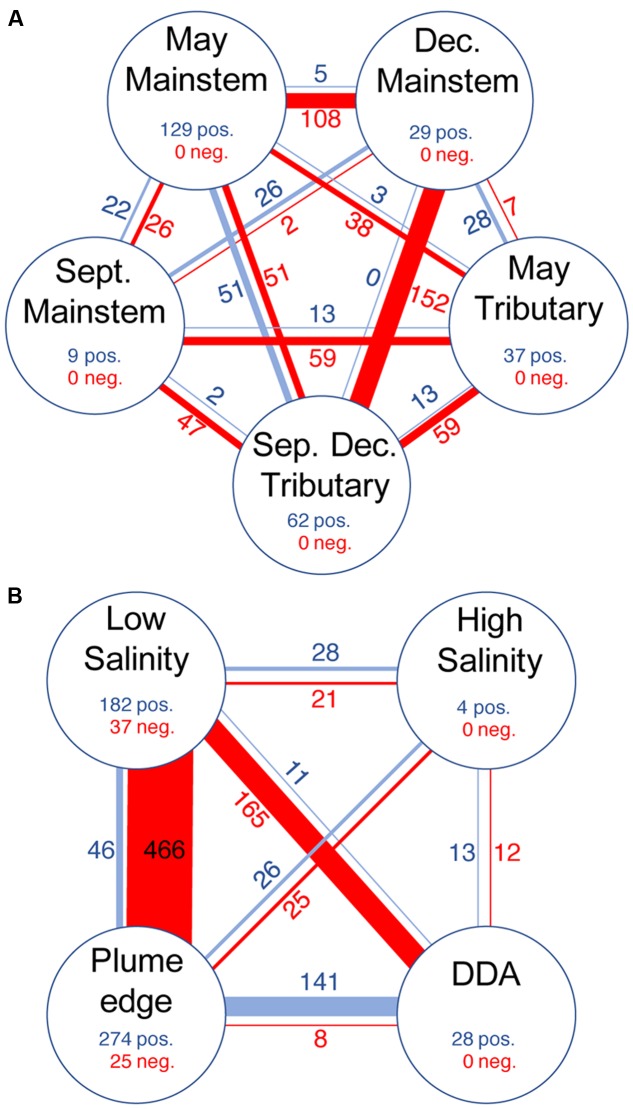
**The number of positive (blue) and negative (red) edges (i.e., correlations) for indicator taxa within co-occurrence networks for** **(A)** all river samples, and **(B)** unfractionated and 0.2–2.0 μm plume samples. Line thickness and numbers indicate the number of edges between node groups. Nodes and edges associated with non-indicator taxa are not shown.

Indicator taxa for river and plume groups accounted for most of the taxa in co-occurrence networks (46 and 89%, respectively), but these taxa varied greatly in the degree to which they showed co-occurrence (positive correlations) and mutual exclusion (negative correlations) (**Table 3**). In the river network, indicator taxa within each indicator group showed strong co-occurrence (266 positive edges and 0 negative edge) (**Figure 4A**), and were much more highly connected for the May and December mainstem than the September mainstem. Indicator taxa in the May and December mainstem groups were also negatively correlated to each other, while September mainstem indicator taxa showed few correlations with themselves or with other indicator groups (**Figure 4A**). Indicator taxa assigned in the May and September-December tributary groups showed mutual exclusion between groups (13 positive edges and 59 negative edges) and with mainstem groups (97 positive edges and 354 negative edges). This mutual exclusion from mainstem groups was stronger for indicator taxa in the September-December tributary group (250 positive edges and 53 negative edges) than the May tributary group (44 positive edges and 104 negative edges) (**Figure 4A**).

**Table 3:** Number of indicator taxa for sample groups, and results of co-occurrence network analyses showing the number of taxa included, the number of positive and negative correlations (edges), and the number of correlations per taxa for each indicator group.

Indicator group	Taxa (*P* < 0.01)	Taxa in network	Positive edges	Negative edges	Edge per taxa
Mainstem May	47	18	460	355	45
Mainstem September	19	10	126	98	22
Mainstem December	63	11	172	370	49
Tributary May	26	11	204	136	31
Tributary September and December	71	21	279	463	35
Non-indicator	3236	82	761	580	16
Total River	3462	153	2002	2002	26

Low salinity	41	28	509	911	51
High salinity	9	9	96	62	18
DDA	51	15	257	194	30
Plume edge	30	26	856	583	55
Non-indicator	1415	47	284	252	11
Total Plume	1496	88	2002	2002	46


Similar to the river network, indicator taxa in the plume network showed a high degree co-occurrence within the plume edge, low salinity, and, to a lesser extent, DDA groups (**Figure 4B** and **Table 3**). Indicator taxa in the low salinity group showed strong mutual exclusion from other indicator groups (85 positive edges and 652 negative edges), with the most mutual exclusion detected with plume edge indicator taxa (46 positive edges and 466 negative edges), and DDA indicator taxa (11 positive edges and 165 negative edges). In contrast, high salinity plume indicators were not strongly correlated with themselves or with taxa in any other group (67 positive edges and 58 negative edges). Indicator taxa in the DDA and plume edge groups showed the strongest co-occurrence between any of the plume indicator groups (141 positive edges and 8 negative edges) (**Figure 4B**).

## Discussion

This first survey of microbial communities along the river-ocean continuum of the largest river in the world revealed that seasonal and spatial patterns were not fundamentally different than those previously observed in smaller rivers (Levine and Crump, 2002; Sekiguchi et al., 2002; Crump and Hobbie, 2005; Winter et al., 2007; Crump et al., 2009), river plumes (King et al., 2013; Mason et al., 2016), and river-ocean continuums (Fortunato et al., 2012; Ortega-Retuerta et al., 2013; Ma et al., 2016), suggesting globally consistent patterns in microbial community composition across river-ocean gradients. We found that river communities varied among tributaries, but mainstem river communities were spatially homogeneous over ∼675 km, and tracked seasonal changes in river discharge. Seasonal shifts in phylogeny were consistent with community variability in other rivers (Fortunato et al., 2013; Ruiz-Gonzalez et al., 2013; Read et al., 2015; Kaevska et al., 2016), and co-occurrence network analyses identified highly interconnected assemblages during high (May) and low (December) discharge periods with a weakly interconnected transitional community in September. These results suggest that the Amazon River has two seasonal microbial communities, as opposed to the three seasonal communities identified for temperate and arctic rivers (Crump et al., 2009; Fortunato et al., 2012). Plume communities showed no seasonal differences and instead varied spatially, loosely tracking salinity. However, salinity only explained a small fraction of the community variability in the plume (**Table 2**), and plume communities were strikingly different in DDA bloom samples than in other high salinity plume samples. These results suggest that although salinity provides physical structure to plume ecosystems, the composition of communities is driven by other environmental and biotic factors including nutrients, DOC chemistry, and phytoplankton community composition (Goes et al., 2014; Medeiros et al., 2015). Co-occurrence networks identified highly interconnected assemblages associated with the low salinity near-shore region, DDA blooms, and the plume edge region, and weakly interconnected assemblages in the high salinity regions, suggesting that the high salinity plume region supports transitional communities that are influenced by mixing of oceanic communities from outside or below the plume, and by species sorting as these communities adapt to local environmental conditions, consistent with Hewson et al. (2006).

### River Communities

Communities in the mainstem Amazon River did not vary spatially over the ∼675 river kilometers between Óbidos and Macapá, suggesting homogeneity in microbially relevant environmental conditions and little influence of tributaries. Similar results were found in long reaches of the lower Changjiang and Danube rivers (Sekiguchi et al., 2002; Winter et al., 2007), but these results contrast with longitudinal surveys of other rivers in which tributary inputs and environmental changes significantly altered microbial communities (Winter et al., 2007; Jackson et al., 2014; Kolmakova et al., 2014; Gladyshev et al., 2015; Read et al., 2015). In the Amazon River, DOM analyses suggest a transition from higher plant-derived DOM to more algal/microbial-derived DOM between Óbidos and Macapá (Seidel et al., 2016), and an associated enhancement in microbial activity and organic matter decomposition rates (Ward et al., 2016). However, the majority of DOM molecular formulae did not change along this reach, suggesting a mixture of compounds resistant to microbial consumption and compounds that are biologically labile but are continuously replenished by autochthonous production and lateral inputs of DOM (Seidel et al., 2016). It is likely that microbial communities at Óbidos, our farthest upstream site, form the final stage of the dynamic equilibrium described by the river continuum concept (Vannote et al., 1980), having already adapted to these new inputs of biologically labile DOM, and it is this community that contributes to changes in DOM composition as it moves from Óbidos to the mouth (Ward et al., 2015).

Amazon River bacterial communities varied seasonally, tracking changes in river discharge and associated environmental conditions. Seasonality is typical of bacterioplankton communities (Crump et al., 2003; Fuhrman et al., 2006; Andersson et al., 2010), and has been documented in several rivers (Crump and Hobbie, 2005; Crump et al., 2009; Staley et al., 2015; Wang et al., 2015). In temperate and Arctic Rivers, seasonal variation correlated with river discharge, rainfall, temperature, nutrient concentration, and organic matter composition (Crump and Hobbie, 2005; Crump et al., 2009; Staley et al., 2015), and in the subtropical Jiulong River correlated with changes in river discharge, temperature, and chlorophyll-*a* (Wang et al., 2015). In the tropical Amazon River mainstem, we found a similar set of environmental factors correlated with microbial communities (**Table 1**). Across all these systems, river discharge appears to be the master variable controlling seasonal changes in microbial communities. Discharge influences the environmental conditions that drive species sorting in microbial communities by controlling the flux of materials (e.g., nutrients and organic matter) and organisms from land and tributaries, and the water residence time available for species sorting to occur. Discharge also influences turbidity and the magnitude of solar insolation, and thus water temperature and phytoplankton production, both of which are potential controls on seasonal patterns in microbial community composition.

The strongest signal of seasonality in river microbial communities was a shift from a high proportion of Betaproteobacteria during high discharge (May) to high proportions of Actinobacteria, Cyanobacteria, and freshwater SAR11 Alphaproteobacteria during low discharge (September and December). A similar pattern was seen in the Columbia River, with more Betaproteobacteria during the spring freshet, and more Actinobacteria and freshwater SAR11 during summer and fall (Fortunato et al., 2013). A study on the effects of impoundment on the Ebro River showed that damming reduced Betaproteobacteria and increased Actinobacteria and Alphaproteobacteria (Ruiz-Gonzalez et al., 2013), suggesting that Betaproteobacteria are favored by the more dynamic conditions of elevated river discharge. This is consistent with a catchment-scale study of the Thames River that linked the development of Actinobacteria communities to water residence time (Read et al., 2015).

In-stream water residence time is negatively related to discharge in rivers (Worrall et al., 2014), and in the lower Amazon River is also modified by tides, which can be detected more than halfway between the mouth and Óbidos (Ward et al., 2016). During low and falling discharge conditions in the Amazon River, DOM was enhanced in compounds containing N and P, likely due to higher contributions of algal biomass from clearwater tributaries such as the Tapajós River (Seidel et al., 2016) and floodplain lakes (Ward et al., 2015). Oxygen saturation was also much higher in September (80%) and December (87%) than in May (average 56% at Macapá), suggesting a seasonal shift in the balance between photoautotrophy and heterotrophy. POC and DOC concentrations at Macapá followed a similar seasonal pattern (Ward et al., 2015), suggesting greater light availability for photoautotrophy during low discharge and greater organic matter for heterotrophy during high discharge. Moreover, bulk respiration rates in the Amazon River are generally highest during low discharge (Benner et al., 1995; Ellis et al., 2012), but the relative decomposition rate of terrestrially derived DOM is highest during high discharge (Ward et al., 2013). Taken together, these observations provide strong evidence that river discharge drives seasonal patterns in river microbial community composition by controlling the ratio of terrestrial to algal-produced DOM, and by controlling the time available for species sorting to produce a microbial community adapted to these DOM conditions.

### Plume Communities

In the Amazon plume, microbial community composition did not vary by season and instead varied with salinity and several co-varying environmental factors. We found that communities in unfractionated samples and in the 0.2–2.0 μm size fraction together formed groups defined by low salinity, high salinity, DDA bloom, and plume edge (**Figure 2**). This contrasts with the Columbia River plume, where seasonality in microbial communities corresponded to seasonal changes in coastal upwelling conditions and to mixing of seasonally varying river communities (Fortunato et al., 2012). The Amazon River plume is much larger and longer-lived than the Columbia River plume (Coles et al., 2013), and river bacteria were almost entirely absent from plume samples (average 0.1% of low salinity group). Also, seasonal changes in environmental conditions (e.g., temperature, sunlight, and upwelling) are smaller in the western tropical Atlantic Ocean compared to the eastern subtropical Pacific Ocean. These factors limit seasonal changes in the environmental conditions experienced by tropical river plume microbial communities and accentuate spatial variability across the salinity gradient that reflects the gradual mixing of river water.

A number of studies show changing community composition along salinity gradients in estuarine and coastal environments (Crump et al., 2004; Fortunato and Crump, 2011; Fortunato et al., 2012; Campbell and Kirchman, 2013). In the Amazon plume, these changes tracked shifts in the composition and dynamics of DOM (Medeiros et al., 2015), and shifts in the dominant phytoplankton groups (Goes et al., 2014). Medeiros et al. (2015) showed that DOM composition varied with salinity due to dilution of riverine DOM, but also showed that deviations from a simple river-ocean mixing model were driven primarily by bacterial transformation of DOM in low salinity plume water, phytoplankton production of new DOM in low and high salinity plume water, and photochemical transformation in water outside the plume. Goes et al. (2014) study of plume phytoplankton identified these same three salinity regions, with a low salinity community of diatoms, cryptophytes and “green water” *Synechococcus* spp., a mesohaline community dominated by DDAs, and an oceanic community of *Trichodesmium* spp. and “blue water” *Synechococcus* spp. We found significant differences in bacterial community composition between all of these regions, and, within the high salinity plume region, communities varied depending on whether they co-occurred with a DDA bloom. These results suggest that although microbial community composition tracks salinity across the entire Amazon River plume, it is more likely that bacterial communities are controlled by the composition of phytoplankton communities and the chemistry of DOM. This conclusion is supported by our correlation analyses (**Table 2**), which show that chlorophyll fluorescence and several other factors contribute significantly to models of bacterial community composition within most regions of the plume.

Low salinity communities correlated most strongly with DOC concentration and phosphate (PO_4_^3-^), but also correlated with several other factors. This community had the highest proportions of Bacteroidetes (Flavobacteriia and Sphingobacteriia) and Gammaproteobacteria, and a recent study demonstrated high gene expression by these taxa in several of these samples (Satinsky et al., 2014a). Bacteroidetes degrade polymers and consume high molecular weight organic matter (Cottrell and Kirchman, 2000; Fernandez-Gomez et al., 2013), and are often prevalent in productive environments such as phytoplankton blooms (Simon et al., 1999) and upwelling zones (Alonso-Saez et al., 2007). Gammaproteobacteria are also typical of phytoplankton blooms (Teeling et al., 2012; Buchan et al., 2014), and, along with Flavobacteriia, were found to produce glycoside hydrolases and other carbohydrate-active enzymes during a diatom bloom in the North Sea (Teeling et al., 2012). These taxa distinguish the low salinity community from the other plume communities (Supplemental Figure S4), and are likely involved in transforming DOM from the river (Medeiros et al., 2015) and from the low salinity community of phytoplankton (Goes et al., 2014).

In contrast, the high salinity, plume edge, and DDA communities had higher proportions of Cyanobacteria (*Synechococcus*, *Prochlorococcus*, and *Richelia*), consistent with a parallel study of phytoplankton community composition (Goes et al., 2014), except that we detected very few *Trichodesmium* spp. High salinity and DDA communities correlated most strongly with bacterial production rate, suggesting that these communities are undergoing species sorting to a different and more active microbial community. Bacterial communities in these groups also had high proportions of Alphaproteobacteria (SAR11 and other Rickettsiales), which are typically associated with oligotrophic conditions (Gilbert et al., 2012; Giovannoni et al., 2014). These results suggest that the high salinity plume environments feature oligotrophic marine communities that are undergoing change to adapt to more nutrient rich and productive conditions.

Diatom-diazotroph assemblage communities had very high proportions of Verrucomicrobia (average 14%) mostly from the family Puniceicoccaceae. Verrucomicrobia are ubiquitous in the world’s oceans, averaging 1.8% of sequences in one global survey, and are more abundant in coastal waters (Freitas et al., 2012). Relatives of Puniceicoccaceae have been isolated from corals (Mavromatis et al., 2010), and aquatic plants including seagrass (Yoon et al., 2007) and duckweed (Matsuzawa et al., 2010). The genome sequence of the Puniceicoccaceae *Coraliomargarita akajimensis* includes genes for many sulfatases, α-L-fucosidases and β-agarases, suggesting that these organisms specialize in the degradation of plant biomass and exudates. Verrucomicrobia were not particularly abundant in the low salinity plume despite high abundance of diatoms (Goes et al., 2014), suggesting that there is something distinctive about the diatoms in DDA blooms that favors the growth of Verrucomicrobia. A metatranscriptomic study showed that Verrucomicrobia maintained some of the highest rates of gene expression (transcripts per gene) among Amazon plume bacteria (Satinsky et al., 2014b), particularly in the particle-attached fraction, further supporting the idea that Verrucomicrobia have a close association with DDAs.

### Particle-Attached Communities

Particle-attached bacteria are often different than free-living bacteria in the plankton of lakes (Riemann and Winding, 2001; Parveen et al., 2011), rivers and estuaries (Crump et al., 1999; Lemke et al., 2009; Pieck et al., 2015), and marine environments (Delong et al., 1993; Kellogg and Deming, 2009). However, in some systems there was very little difference between size fractions including the Mackenzie River (Ortega-Retuerta et al., 2013), Pearl River (Zhang et al., 2016), and the Sacramento River and upper San Francisco Bay (Hollibaugh et al., 2000). Particle-attached and free-living bacterial communities in the Amazon River were very similar based on 16S rRNA gene amplicons, and showed small differences based on metagenomic and metatranscriptomic sequencing (Satinsky et al., 2015). This suggests rapid exchange of organisms between these two environments, and no difference in growth conditions that might be caused by phytoplankton aggregate formation (Grossart et al., 2006) or inputs of fresh allochthonous POM.

In contrast, plume microbial communities were very different in the two size fractions at most stations due primarily to reduced proportions of *Synechococcus*, *Prochlorococcus*, and SAR11 Alphaproteobacteria, and elevated proportions of Gammaproteobacteria, Bacteroidetes, and Planctomycetes in the >0.2 μm size fractions. At low salinity station 10, sampled in June 2010, the gene expression patterns of particle-attached microbes were strikingly different than those of free-living bacteria, with elevated expression of genes for sulfur cycling, aromatic compound degradation and synthesis of vitamins (Satinsky et al., 2014a). In addition, higher concentrations of saccharides (short-lived compounds) were observed in POC collected at station 10 compared to other plume stations, suggesting a major input of phytoplankton organic matter (Medeiros et al., 2015). This suggests that growth conditions on plume particles are different than those for free-living bacteria, and those differences drive changes in microbial community composition.

### Co-occurrence Networks

We combined the two independent analysis techniques of co-occurrence networks and indicator analysis to differentiate between stable and transitional microbial communities, and to infer potential microbial interactions across the Amazon River continuum. Indicator taxa in the river network co-occurred strongly within the May and December mainstem indicator groups, and these groups were strongly negatively correlated to each other (**Figure 4A** and **Table 3**), suggesting that these taxa are the strongest indicators of high discharge (May) and low discharge (December) conditions in the Amazon River. In contrast, September mainstem indicator taxa showed few correlations (positive or negative), suggesting that September indicator taxa represent a transitional community between the presumably stable communities that form during high and low discharge. Similarly, tributary indicator taxa showed strong co-occurrence within seasonal indicator groups and mutual exclusion between groups, demonstrating seasonality in tributary communities. Correlations between mainstem and tributary indicators were strongly negative during December but not during May, suggesting that tributary communities are more highly represented in the mainstem river during the high discharge season.

Plume indicators co-occurred strongly within the low salinity and plume edge groups, and these groups were strongly negatively correlated (**Figure 4B** and **Table 3**). In contrast, high salinity plume indicators showed few correlations, suggesting that the high salinity plume contains a transitional community between the low salinity plume and open ocean. DDA indicators were positively correlated with themselves and with plume edge indicators, and negatively correlated with low salinity indicators (**Figure 4B**) suggesting that DDA communities develop from open ocean communities following DDA bloom development, and are very different than the communities that develop associated with non-DDA phytoplankton blooms at lower salinities. These results suggest that river plumes host transitional mixtures of organisms that grow into more stable communities under elevated growth conditions (in this case DDA and low salinity phytoplankton blooms), and that the composition of these communities depends not on salinity but instead on factors that drive those high growth conditions.

### Comparison with Other Systems

**FIGURE 5:**
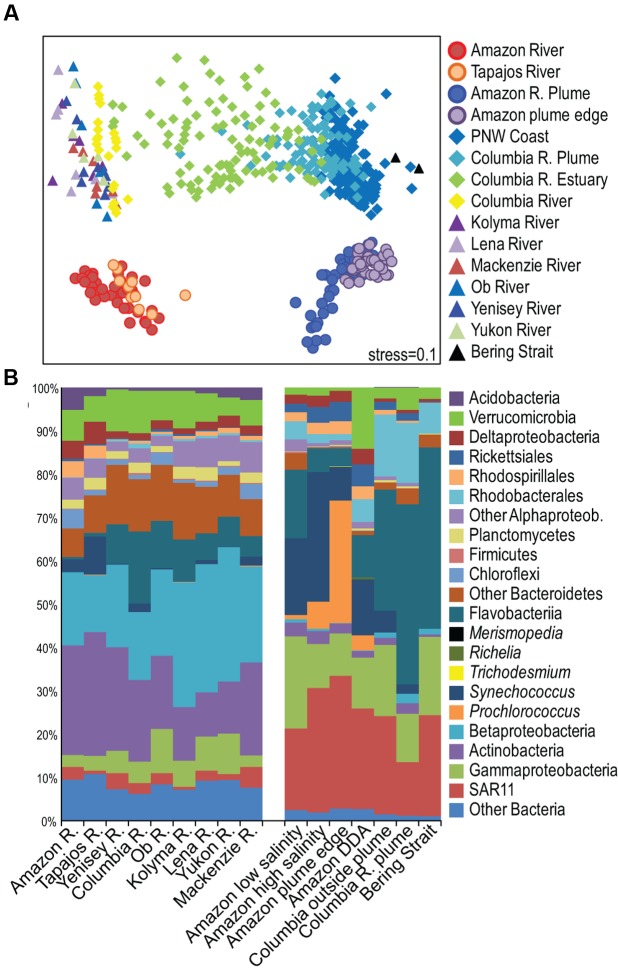
**(A)** Multidimensional Scaling (MDS) diagram of Bray–Curtis similarity among bacterial communities in all samples collected for this study, communities from the Columbia River, estuary, plume, and surface ocean (Fortunato et al., 2013), and communities from six arctic rivers (Crump et al., 2009). **(B)** Average taxonomic diversity of bacterial communities in river, plume, and coastal ocean environments.

Few studies describe microbial community composition across the full continuum from river to open ocean, and no studies have compared these continuums from different systems. Our comparison of tropical, temperate, and arctic rivers showed that Amazon river, plume, and coastal ocean bacterial communities were distinct from communities in the Columbia River system and from communities in six large arctic rivers (Yukon, Yenisei, Ob, Lena, Kolyma, and Mackenzie) (**Figure 5A** and Supplemental Figure S6). Actinobacteria and Betaproteobacteria dominated all river communities, but the Amazon and Tapajós rivers had higher proportions of Chloroflexi, Acidobacteria, Nitrospirae, and Deltaproteobacteria than other rivers, and a much lower proportion of Flavobacteriia and other Bacteroidetes (**Figure 5B**). Bacteroidetes were detected in the tropical Paraná River, but these taxa did not include Flavobacteriia (Lemke et al., 2009), and Bacteroidetes were not detected in a small survey of bacteria in the tropical Solimões and Negro River tributaries of the Amazon (Peixoto et al., 2011). A small fraction of sequences from an Amazon River metagenome mapped to Bacteroidetes, but it is not clear whether those taxa were Flavobacteriia (Ghai et al., 2011). Among the other rivers in our study, Flavobacteriia was lowest in the highly turbid Mackenzie River where one earlier study found very few Flavobacteriia (Ortega-Retuerta et al., 2013). Freshwater Flavobacteriia abundance has been linked to phytoplankton production in autotrophic systems (Eiler and Bertilsson, 2007; Newton et al., 2011), suggesting that their absence from the Amazon results from high turbidity and low photoautotrophy rather than from its location in the tropics.

Amazon plume communities were different than Columbia plume and Bering Strait communities, and again one of the most important differences was low Flavobacteriia in the Amazon, along with a higher proportion of Cyanobacteria and Deltaproteobacteria (**Figure 5B** and Supplemental Figure S6). Flavobacteriia were present in the Amazon plume, particularly at low salinities, and were active based on metatranscriptomic sequencing (Satinsky et al., 2014b). However, their abundance was much lower than in the Columbia River plume and the Bering Strait where they accounted for nearly 40% of 16S rRNA genes (**Figure 5B**). Marine bacterial taxa exhibit latitudinal ranges (Amend et al., 2013), and one global survey of ocean bacteria found a negative relationship between Bacteroidetes richness and temperature (Pommier et al., 2007). However, Bacteroidetes are often a significant fraction of tropical freshwater and marine communities (Rusch et al., 2007; Silveira et al., 2011; Lau et al., 2013; Tada and Suzuki, 2016), and Flavobacteriia accounted for a relatively high proportion of communities in low salinity plume and DDA blooms where diatoms were more abundant (Goes et al., 2014). In fact, Flavobacteriia were the most strongly networked indicator taxa in the low salinity communities (Supplemental Table S4), suggesting that Flavobacteriia are central members of bacterial communities that develop in productive regions of river plumes and may serve as global indicators of eutrophic conditions in coastal zones.

This cross-system comparison suggests globally consistent patterns and controls on the composition of microbial communities across river-ocean continuums. At the phylum/class level, river microbial communities were fairly similar and appeared to vary with river turbidity, supporting the idea that river microbial community composition depends on the ratio of allochthonous vs. autochthonous DOM. Plume communities tracked salinity in both the Amazon and Columbia plumes, and both systems were strongly influenced by mixing with coastal ocean communities, which differed greatly in the two regions. However, productive regions of both systems developed abundant populations of Bacteroidetes, including Flavobacteriia, suggesting that although plume communities are structured by salinity through buoyancy and mixing, the composition of plume-specific communities is controlled by factors unique to productive regions including nutrients, phytoplankton community composition, and DOM chemistry.

## Author Contributions

MD for laboratory analysis, data analysis, intellectual contributions, and manuscript preparation. PY for project leadership, intellectual contributions, sample collection, and laboratory analysis. MM for project leadership, intellectual contributions, and manuscript preparation. VC for sample collection, laboratory analysis, and intellectual contributions. CF for sample collection, laboratory analysis, intellectual contributions, and manuscript preparation. AK for project leadership, sample collection, and intellectual contributions. PM for laboratory analysis, intellectual contributions, and manuscript preparation. JP for data analysis, intellectual contributions, and manuscript preparation. JR for project leadership, sample collection, and intellectual contributions. BS for sample collection, intellectual contributions, and manuscript preparation. HS for sample collection, laboratory analysis, and manuscript preparation. NW for sample collection, laboratory analysis, intellectual contributions, and manuscript preparation. BC for project leadership, intellectual contributions, data analysis, and manuscript preparation.

## Conflict of Interest Statement

The authors declare that the research was conducted in the absence of any commercial or financial relationships that could be construed as a potential conflict of interest.

## Footnotes

1 http://engencore.sc.edu/

2 www.ncbi.nlm.nih.gov

3 www.cytoscape.org
