# Supplementary material for: Bacterial Biogeography across the Amazon River-Ocean Continuum
Source: Front Microbiol. 2017 May 23;8:882. doi: 10.3389/fmicb.2017.00882 (PMC5440517; doi:10.3389/fmicb.2017.00882)

Bacterial biogeography across the Amazon river-ocean continuum

Mary Doherty[1], Patricia L. Yager[2], Mary Ann Moran[2], Victoria J. Coles[1], Caroline S. Fortunato[3], Alex V. Krusche[4], Patricia M. Medeiros[2], Jérôme P. Payet[5], Jeffrey E. Richey[6], Brandon M. Satinsky[7], Henrique O. Sawakuchi[4], Nicholas D. Ward[8], Byron C. Crump[5*]

1. University of Maryland Center for Environmental Science, Cambridge, MD 21613
2. University of Georgia, Athens, GA 30602
3. Marine Biological Laboratory, Woods Hole, MA 02543
4. University of Sao Paulo, Av. Centenario, 303, Piracicaba, SP 13400-970, Brazil
5. Oregon State University, Corvallis, OR 97331
6. University of Washington, Seattle, WA, USA 98195
7. Massachusetts Institute of Technology, Cambridge, MA 02139
8. Pacific Northwest National Laboratory, Sequim, WA, USA 98382

Supplemental Tables and Figures

Supplemental Table S1. Sampling stations in the Amazon River and plume.

Station	River & Plume location	latitude (deg.)	longitude (deg.)
OB_L	Óbidos Left	-55.5101	-1.9251
OB_C	Óbidos Center	-55.5257	-1.9190
OB_R	Óbidos Right	-55.5257	-1.9295
TAP	Tapajós Center	-55.0075	-2.4844
MCP_N_L	Macapá North Left	-51.0810	-0.0567
MCP_N_C	Macapá North Center	-51.0514	-0.0839
MCP_N_R	Macapá North Right	-51.0215	-0.1240
MCP_S_L	Macapá South Left	-50.6337	-0.1325
MCP_S_C	Macapá South Center	-50.6226	-0.1569
MCP_S_R	Macapá South Right	-50.6100	-0.1792
BLM_L	Belém Left	-48.9125	-1.5097
BLM_C	Belém Center	-48.9180	-1.5194
BLM_R	Belém Right	-48.9076	-1.5649
1_2010	high salinity plume	11.5644	-56.7984
2_day_2010	DDA	10.2885	-54.5120
2_night_2010	DDA	10.2908	-54.5113
3_2010	high salinity plume	7.2875	-53.0005
4_2010	low salinity plume	5.9427	-51.4953
4_2010	below plume	5.9427	-51.4953
5_2010	plume edge	6.8137	-49.9801
6_2010	plume edge	6.8177	-47.6341
7_2010	plume edge	7.0014	-45.0207
8_2010	plume edge	4.3489	-46.8504
9_2010	high salinity plume	6.0018	-50.8389
10_2010	low salinity plume	4.8818	-51.3608
11_2010	low salinity plume	5.4919	-51.4975
13_2010	low salinity plume	6.5120	-51.3370
16_2010	low salinity plume	6.6932	-51.7774
19_2010	plume edge	8.2961	-50.7599
20_2010	plume edge	9.9577	-50.0191
21_2010	plume edge	9.7674	-51.6936
23_2010	high salinity plume	10.6822	-54.4213
23_2010	below plume	10.6822	-54.4213
25_2010	DDA	11.3123	-56.4267
27_2010	plume edge	12.4145	-52.2197
1_2011	plume edge	11.6333	-56.8833
2_2011	plume edge	10.8500	-55.0500
3_2011	plume edge	10.2667	-54.5333
4_2011	plume edge	8.0000	-52.9667
6_2011	low salinity plume	6.8333	-51.3333
7_2011	low salinity plume	6.8667	-50.7833
8_2011	high salinity plume	6.9167	-49.9500
9_2011	plume edge	6.6500	-48.5667
11_2011	low salinity plume	5.1500	-51.2333
12_2011	low salinity plume	6.2500	-51.3833
13_2011	high salinity plume	7.9500	-51.3333
19_2011	plume edge	6.8167	-46.0333
21_2011	plume edge	5.4333	-44.6333
22_2011	plume edge	7.0000	-44.8500
23_2011	plume edge	8.5833	-44.9000
25_2011	plume edge	8.4667	-48.0500
26_2011	plume edge	7.4667	-48.9167
27_2011	plume edge	8.0833	-49.5333
28_2011	high salinity plume	9.0000	-50.0000
29_2011	high salinity plume	9.5167	-50.4000
32_2011	plume edge	10.6667	-52.9833

Table S2. Environmental data for samples collected from the Amazon River. Discharge was calculated as the sum of discharge of the mainstem river at Óbidos plus the Tapajós, Xingu, and Tocantins Rivers on those sampling dates using data from Ward et al. (2015).

Station	date (local)	Amazon R. Discharge (m^3/s)	Sampling depth (m)	sample types	Sample numbers	Conduc-tivity (µSi/cm)	Temp-erature (°C)	pH (pH)	O2 (mg/L)	DOC (µM)	DIC (µM)	CO2 (µM)	Nitrite (µM)	Nitrate (µM)	Ammo-nium (µM)	DIN (mg/L)	DON (mg/L)	Cl (µM)	SO4 (µM)	PO4 (µM)	Na (µM)	K (µM)	Mg (µM)	Ca (µM)	Si (mg/L)	TDN (mg/L)
MCP_N_C	16-Sep-2010	134500	17	whole	142	52.1	31.3	6.8	5.1	403	210	54	0.85	9.6	0.52	0.16	0.09	63	42	0.26	40	16	25	93	3.3	0.25
				0.2-2.0um	145																					
				>2.0um	143																					
MCP_N_L	16-Sep-2010	134500	17	whole	150	46.5	30.0	6.8	5.9	368	206	54	0.87	6.8	0.00	0.10	0.18	75	37	0.55	41	16	25	92	3.3	0.28
MCP_S_R	18-Sep-2010	134500	14	whole	152	46.8	30.3	7.0	5.5	360	207	38	0.70	1.3	0.00	0.03	0.23	61	39	0.41	38	16	24	92	3.4	0.26
MCP_S_C	18-Sep-2010	134500	13	whole	154																					
OB_C	23-Sep-2010	134500	27	whole	156	58.6	30.8	6.9	5.1	339	221	221	0.70	13.5	1.52	0.24	0.05	73	31	0.31	45	15	32	125	3.9	0.29
OB_R	23-Sep-2010	134500	22	whole	158	76.1	31.1	6.8	5.4	366	248	248	0.75	12.8	0.55	0.20	0.12	71	32	0.32	45	14	35	127	4.0	0.32
OB_L	23-Sep-2010	134500	30	whole	160	48.3	30.9	6.8	5.6	339	253	253	0.42	10.2	1.32	0.19	0.10	67	30	0.30	40	15	30	114	3.9	0.28
TAP	23-Sep-2010	134500	15	whole	162	21.5	30.8	7.2	6.9	120	110	110	0.52	1.7	0.87	0.06	0.02	14	4	0.40	13	10	20	25	4.4	0.07
BLM_L	7-Dec-2010	89800	5.5	whole	163	54.1	30.3	7.1	5.7	426	291	42	1.20	3.9	3.74	0.19	0.13	101	21	0.25	86	43	76	113	0.8	0.32
TAP	14-Dec-2010	89800	16	whole	173	14.4	30.6	6.8	7.7	204	75	75	1.35	2.9	3.59	0.17	0.04	20	10	0.24	20	8	24	27	4.1	0.22
TAP	14-Dec-2010	89800	0.5	whole	175	15.8	32.0	7.1	5.4	.	104	104	2.00	4.3	1.85	0.14		37	12	0.20	53	17	16	24	4.0	0.09
MCP_N_R	1-Dec-2010	89800	12	whole	177	89.4	31.0	7.5	6.4	224	490	29	0.20	18.2	0.00	0.25	0.10	110	59	0.59	148	35	87	283	5.6	0.36
MCP_N_C	1-Dec-2010	89800	14	whole	179	83.0	30.5	7.5	6.7	293	476	29	0.27	18.3	0.15	0.26	0.10	105	42	0.57	104	23	61	193	4.0	0.36
MCP_N_L	1-Dec-2010	89800	15	whole	181	83.1	30.5	7.5	6.3	237	487	32	0.32	18.3	1.47	0.30	0.07	109	42	0.57	115	23	61	219	4.0	0.37
MCP_S_R	3-Dec-2010	89800	12	whole	183	85.5	30.8	7.6	6.4	238	494	26	0.32	13.3	0.00	0.19	0.16	117	59	0.32	155	33	83	178	5.1	0.34
MCP_S_L	3-Dec-2010	89800	2	whole	185	89.3	30.8	7.5	6.3	233	495	30	0.37	15.7	0.00	0.22	0.12	124	46	0.17	119	25	64	226	4.1	0.34
MCP_N_L	7-May-2011	334400	15	whole	193	56.3	28.6	6.8	4.4	478	435	117	1.45	17.0	1.37	0.29	0.06	91	39	0.63	60	28	55	174	4.5	0.36
MCP_N_C	7-May-2011	334400	14	whole	195	61.5	29.0	6.8	4.5	476	403	110	1.60	12.9	0.00	0.20	0.15	95	40	0.52	60	29	55	173	4.4	0.34
				0.2-2.0um	189																					
				>2.0um	190																					
MCP_N_R	7-May-2011	334400	12	whole	197	59.1	28.9	6.7	4.0	483	420	124	1.57	15.9	0.00	0.24	0.13	96	44	0.54	64	29	58	186	4.3	0.37
MCP_S_L	9-May-2011	334400	20	whole	208	55.3	29.0	6.8	4.4	445	381	100	1.32	11.2	0.00	0.17	0.18	106	37	0.58	58	30	51	154	4.5	0.35
MCP_S_C	9-May-2011	334400	10	whole	204	55.3	28.8	6.7	4.3	451	408	115	1.37	10.9	0.00	0.17	0.18	66	38	0.46	53	29	52	166	4.5	0.35
		334400		whole	210																					
		334400		0.2-2.0um	203																					
MCP_S_R	9-May-2011	334400	12	whole	212	51.7	28.6	6.8	4.4	471	408	108	1.55	10.4	0.00	0.16	0.18	66	36	0.42	53	30	52	162	4.4	0.34
BLM_L	13-May-2011	334400	35	whole	223	34.9	29.1	7.5	5.3	599	214	12	2.32	5.8	1.02	0.14	0.25	73	18	0.41	62	31	39	68	3.9	0.39
BLM_C	13-May-2011	334400	19	whole	219	37.1	29.3	7.4	5.6	381	294	25	1.62	5.6	5.77	0.28	0.11	58	10	0.41	67	38	53	70	3.9	0.39
				whole	225																					
				0.2-2.0um	217																					
				>2.0um	218																					
BLM_R	14-May-2011	334400	2	whole	229																					
OB_L	19-May-2011	334400	27.5	whole	240	56.9	27.9	6.6	3.2	1607	390	390	5.42	12.9	0.00	0.24	0.37	58	72	0.27	79	25	62	142	6.9	0.61
OB_C	19-May-2011	334400	33	whole	236	56.4	28.4	6.6	3.2	2659	357	357	5.74	13.6	0.90	0.28	1.24	51	92	0.28	73	8	64	156	8.8	1.53
				whole	242																					
				0.2-2.0um	234																					
				>2.0um	235																					
OB_R	19-May-2011	334400	24	whole	246	68.5	28.3	6.7	3.0	3310	369	369	4.92	9.1	0.00	0.18	0.28	51	65	0.26	30	7	61	147	6.2	0.47
TAP	20-May-2011	334400	15	whole	253	16.8	29.5	6.5	6.0	1990	107	107	4.74	8.8	0.00	0.18	3.37	18	13	0.31	13	6	28	43	2.2	3.55
				whole	257																					
				0.2-2.0um	251																					
				>2.0um	252																					
TAP	20-May-2011	334400	1	whole	268	15.9	28.9	6.5	6.1	1656	106	106	3.69	8.1	2.77	0.25	1.36	17	11	0.17	13	13	22	18	1.9	1.61
				0.2-2.0um	265																					
				>2.0um	266																					

Table S3. Environmental data for samples from the Amazon River plume.

Station	Date	Sampling depth (m)	Sample types	Sample #s	Sal-inity (PSU)	Temp-erature (°C)	O2 (µmol/kg)	DOC (µM)	Nitrate +Nitrite (µM)	PN (µM)	POC (µM)	Phosphate (µM)	Silica (µM)	Fluor-escence (mg/m^3)	Chlorophyll a (µg/L)	Bacteria Count (cells/ml)	Bacteria Production (pmol leu/L/hr)
1_2010	23-May-2010	3.85	whole	8	32.0	28.8	188.3	101.0	0.014	1.24	8.3	0.05	10.4	0.09	0.29	447508	34.1
			0.2-2.0um	2,3													
			>2.0um	6													
2_day_2010	24-May-2010	4.24	whole	16	32.5	28.8	199.7	98.0	0.000	0.95	9.1	0.09	5.9	0.56	3.25	615538	112.5
			0.2-2.0um	11,12													
			>2.0um	14,15													
2_night_2010	24-May-2010	4.24	whole	24	32.5	28.8	199.7	98.0	0.000	0.95	9.1	0.09	5.9	0.56	3.25	615538	112.5
			0.2-2.0um	18,19													
			>2.0um	21													
3_2010	26-May-2010	3.76	whole	32	30.8	28.9	188.3	101.0	0.000	0.47	3.8	0.19	17.0	0.28	0.58	583763	139.7
			0.2-2.0um	27,28													
			>2.0um	29,30													
4_2010	27-May-2010	3.58	whole	40	22.2	29.1	234.8	157.0	0.374	0.75	30.3	0.30	39.8	1.38	8.33	1610214	610.2
			0.2-2.0um	34,35													
			>2.0um	38,39													
4_2010	27-May-2010	21.63	whole	42	35.8	28.9	183.4					0.03	1.8	0.21	0.55		
5_2010	28-May-2010	3.71	whole	50	35.2	28.7	186.4	87.0	0.000	0.15	2.1	0.00	2.8	0.05	0.27	589328	37.0
			0.2-2.0um	44,45													
			>2.0um	48,49													
6_2010	29-May-2010	3.77	whole	52	35.6	28.7	187.0	99.0	0.025	0.24	2.0	0.03	0.8	0.02	0.17	500297	27.5
7_2010	31-May-2010	3.94	whole	60	35.9	28.1	187.5	80.0	0.056	0.41	2.7	0.05	0.8	0.05	0.32	567027	19.0
			0.2-2.0um	54,55													
			>2.0um	57,59													
8_2010	1-Jun-2010	3.68	whole	62	35.6	28.7	187.4	86.9	0.000	0.32	2.4	0.00	0.8	0.02	0.24	363533	16.5
9_2010	3-Jun-2010	3.60	whole	70	33.2	29.4	183.4	98.0	0.000	0.78	6.4	0.06	5.6	0.21	0.68	627956	66.1
			0.2-2.0um	64,65													
			>2.0um	68,69													
10_2010	5-Jun-2010	4.26	whole	78	22.6	29.4	251.6	97.0	0.188	7.46	70.2	0.39	38.5	7.25	33.26	1845807	328.0
			0.2-2.0um	72,73,74													
			>2.0um	75,76,77													
11_2010	6-Jun-2010	3.20	whole	86	22.4	29.6	275.5	125.0	0.000	2.52	35.7	0.31	35.0	3.08	9.00	839735	324.8
			0.2-2.0um	80,82													
13_2010	7-Jun-2010	3.73	whole	88	21.9	30.0	204.6		0.000	0.31	2.1	0.38	40.2	0.37			
16_2010	8-Jun-2010	3.94	whole	90	20.9	29.4	212.3	136.8	0.000	0.81	8.3	0.59	51.6	0.55	1.12	595327	287.6
19_2010	11-Jun-2010	3.42	whole	92	34.9	28.7	188.8	94.9	0.000	0.53	4.5	0.07	2.4	0.06	0.34	677273	51.3
20_2010	12-Jun-2010	3.50	whole	94	35.2	28.5	187.8	87.1	0.000	0.42	2.8	0.08	1.1	0.03	0.19	568298	32.9
21_2010	13-Jun-2010	3.90	whole	102	33.7	28.7	187.2	89.6	0.032	0.43	2.9	0.12	5.7	0.05	0.16	561613	24.4
			0.2-2.0um	97													
			>2.0um	101													
23_2010	16-Jun-2010	3.64	whole	111	26.5	29.2	194.2	115.9	0.000	1.03	5.7	0.29	25.7	0.16	0.15	737040	79.8
			0.2-2.0um	104, 106													
			>2.0um	109													
23_2010	16-Jun-2010	37.80	whole	117	36.2	28.2	183.1	71.1	0.000	1.11	4.3	0.09	1.1	0.25	0.48	558424	11.3
			0.2-2.0um	113													
			>2.0um	116													
25_2010	18-Jun-2010	3.93	whole	125	31.9	29.2	213.8	108.0	0.000	1.49	19.5	0.00	3.4	0.99	5.25	667527	78.5
			0.2-2.0um	119, 120													
			>2.0um	122, 123													
25_2010	18-Jun-2010	~15m	0.2-2.0um	127, 128													
			>2.0um	129													
27_2010	21-Jun-2010	3.89	whole	137	36.0	28.4	188.4	88.0	0.000	0.31	2.7	0.52	1.2	0.01	0.13	616241	16.1
			0.2-2.0um	131, 132													
			>2.0um	135													
1_2011	5-Sep-2011	2.85	whole	272	32.5	30.3	194.2	78.1	0.930	0.49	3.1	0.35	10.8	0.09	0.17	663315	2.8
2_2011	6-Sep-2011	2.41	0.2µm	277	34.2	29.6	193.3	71.8	0.802	0.54	2.9	0.13	5.5	0.09	0.07	563348	5.7
2_2011	6-Sep-2011	2.50	whole	280	34.1	29.8	194.2	71.8	0.678	0.37	2.5	0.12	5.4	0.08	0.07	477907	5.7
3_2011	7-Sep-2011	2.49	0.2µm	285	34.5	29.1	194.8	64.4	0.309	0.52	3.0	0.11	6.4	0.09	0.13	615569	7.7
3_2011	7-Sep-2011	2.45	whole	288	34.5	29.2	195.0	64.4	0.122	0.42	2.9	0.12	6.3	0.08	0.13	615569	7.7
4_2011	8-Sep-2011	2.29	whole	290	34.2	29.9	189.7	70.2		0.45	3.5			0.11	0.07	1459956	29.3
6_2011	10-Sep-2011	2.87	0.2µm	297	27.6	28.5	235.1	94.9	0.271	1.64	15.5	0.18	20.4	0.20	0.60	1236503	293.0
6_2011	10-Sep-2011	3.57	whole	300	27.9	28.5	233.9	94.9	0.109	1.11	10.6	0.40	24.4	0.20	0.60	1236503	293.0
7_2011	11-Sep-2011	2.08	whole	302	24.2	28.5	244.0	122.4	0.381	0.69	6.3	0.72	36.5	0.30	1.42	842460	
8_2011	12-Sep-2011	2.70	whole	310	33.7	28.4	198.3	78.2	0.000	0.58	3.1	0.41	8.3	0.10	0.15	681208	5.0
9_2011	14-Sep-2011	2.83	0.2µm	315	35.2	28.6	194.9	75.4	0.111	0.49	2.7	0.13	5.5	0.09	0.15	525981	1.8
9_2011	14-Sep-2011	2.20	whole	318	34.7	28.7	195.2	75.4	0.168	0.32	2.3	0.13	5.4	0.09	0.15	525981	1.8
11_2011	16-Sep-2011	2.03	whole	326	29.5	28.5	251.7	86.6	0.000	2.17	11.9	0.79	29.2	0.45	2.39	1988204	306.3
12_2011	18-Sep-2011	2.20	whole	328	23.9	29.2	279.9	91.0	0.000	1.03	9.1	0.70	42.1	0.22	0.67	2167983	351.1
13_2011	19-Sep-2011	2.31	0.2µm	333	30.6	29.0	203.6	80.3	0.000	0.66	4.3	0.40	22.1	0.12	0.20	1129798	87.8
13_2011	19-Sep-2011	2.63	whole	336	31.0	29.3	204.0	80.3	0.363	0.61	4.0	0.59	17.7	0.11	0.20	1129798	87.8
13_2011	20-Sep-2011	2.13	whole	338	35.2	28.4	195.0	57.1	0.000	0.42	2.5	0.19	6.0	0.17		541013	87.8
13_2011	21-Sep-2011	2.55	whole	340	27.5	29.8	208.1	0.0	0.000	0.73	4.7	0.68	34.1	0.17	0.02		87.8
19_2011	23-Sep-2011	2.83	0.2µm	347	31.1	29.2	195.5	71.8	0.000	0.49	2.9	0.46	14.1	0.10	0.16	429553	38.8
19_2011	23-Sep-2011	2.77	whole	350	31.1	29.2	195.7	71.8	0.060	0.38	2.5	0.29	13.1	0.10	0.16	429553	38.8
21_2011	25-Sep-2011	2.78	0.2µm	357	33.3	29.6	193.8	59.7	0.000	0.38	2.5	0.14	7.2	0.10	0.16	534954	40.0
22_2011	26-Sep-2011	2.05	whole	362	31.6	29.4	196.0	68.6	0.000	0.30	2.1	0.14	8.4	0.09	0.10	508330	
23_2011	27-Sep-2011	1.91	0.2µm	365	32.7	29.7	193.1	70.7	0.000	0.28	1.9	0.16	7.1	0.09		1126851	27.5
23_2011	27-Sep-2011	1.99	whole	367	32.7	29.7	193.1	70.7	0.000	0.28	1.9	0.16	7.1	0.09		1126851	27.5
25_2011	29-Sep-2011	2.36	0.2µm	372	31.4	30.0	194.9	60.3	0.000	0.30	1.9	0.25	15.0	0.10	0.15	667505	53.4
25_2011	29-Sep-2011	2.74	whole	375	31.4	30.0	194.7	60.3	0.000	0.32	2.3	0.25	15.3	0.10	0.15	667505	53.4
26_2011	30-Sep-2011	2.60	whole	377	36.1	28.7	193.4	61.9	0.000			0.05	0.9	0.09	0.14	635664	
26_2011	30-Sep-2011	2.18	whole	379	34.4	28.8	196.3	66.4	0.000			0.11	5.4	0.10	0.14	685831	
27_2011	2-Oct-2011	2.98	whole	381	36.1	28.3	193.9	0.0	0.021			0.29	0.8	0.11	0.09		
28_2011	3-Oct-2011	2.27	whole	383	32.6	29.5	193.9	63.6	0.000			0.21	11.1	0.18	0.21	848551	28.7
29_2011	4-Oct-2011	2.96	0.2µm	388	32.3	29.3	192.9	67.1	0.000			0.20	11.8	0.11	0.27	857945	47.9
29_2011	4-Oct-2011	2.96	whole	391	32.3	29.3	192.9	67.1	0.000			0.20	11.8	0.11	0.27	857945	47.9
32_2011	6-Oct-2011	2.09	2.0µm	397	34.3	29.0	192.4	62.5	0.118			0.13	4.9	0.09	0.12	1304305	5.0
32_2011	6-Oct-2011	2.09	whole	399	34.3	29.0	192.4	62.5	0.118			0.13	4.9	0.09	0.12	1304305	24.0

Supplemental Table S4. Significant indicator taxa (p<0.01) for indicator analyses of river samples, plume whole water & <0.2um samples, and plume >2.0um samples.

Sample Group	Indicator group (p<0.01)	OTU#	Indicator value	p value	Average abundance in Sample group	Average abundance in Indicator group	SILVA taxon	FW_clade
River	Mainstem May	7201	0.4693	0.001	1.86%	3.47%	Actinobacteria	Actinobacteria_acI
River	Mainstem May	994	0.5446	0.002	0.96%	2.75%	Betaproteobacteria	
River	Mainstem May	3819	0.3383	0.003	1.25%	1.83%	Betaproteobacteria	Betaproteobacteria_betI
River	Mainstem May	6840	0.5121	0.001	0.87%	1.74%	Betaproteobacteria	Betaproteobacteria_betII
River	Mainstem May	7503	0.8845	0.001	0.43%	1.23%	Betaproteobacteria	Betaproteobacteria_betII
River	Mainstem May	9188	0.5189	0.001	0.58%	1.16%	Betaproteobacteria	Betaproteobacteria_betIV
River	Mainstem May	4375	0.9545	0.001	0.36%	1.07%	Other Bacteria	
River	Mainstem May	8628	0.5167	0.001	0.50%	0.97%	Chloroflexi	
River	Mainstem May	13210	0.3584	0.009	0.45%	0.76%	Other Bacteria	
River	Mainstem May	4005	0.3677	0.004	0.38%	0.59%	Betaproteobacteria	
River	Mainstem May	8378	0.5127	0.001	0.30%	0.58%	Betaproteobacteria	Betaproteobacteria_betI
River	Mainstem May	11525	0.6285	0.001	0.26%	0.58%	Actinobacteria	Actinobacteria_acI
River	Mainstem May	12784	0.6110	0.001	0.26%	0.57%	Actinobacteria	Actinobacteria_acSTL
River	Mainstem May	3767	0.5089	0.001	0.27%	0.54%	Gammaproteobacteria	
River	Mainstem May	2888	0.3409	0.009	0.37%	0.53%	Actinobacteria	Actinobacteria_acIV
River	Mainstem Sept.	3961	0.3982	0.001	0.95%	1.79%	Chloroflexi	
River	Mainstem Sept.	11042	0.3848	0.001	0.44%	0.75%	Verrucomicrobia	
River	Mainstem Sept.	12452	0.5587	0.001	0.18%	0.59%	Rhodospirillales	
River	Mainstem Sept.	4286	0.5885	0.001	0.19%	0.55%	Rhodospirillales	
River	Mainstem Sept.	3314	0.5625	0.001	0.18%	0.53%	Planctomycetes	
River	Mainstem Dec.	87	0.5670	0.001	1.31%	4.43%	Actinobacteria	Actinobacteria_acIV
River	Mainstem Dec.	3762	0.5784	0.001	0.89%	2.87%	Actinobacteria	
River	Mainstem Dec.	3340	0.5669	0.001	0.75%	2.48%	Actinobacteria	Actinobacteria_acI
River	Mainstem Dec.	6516	0.9264	0.001	0.34%	2.29%	Actinobacteria	Actinobacteria_acIV
River	Mainstem Dec.	7812	0.4773	0.001	0.84%	2.25%	Actinobacteria	Actinobacteria_acI
River	Mainstem Dec.	4261	0.7119	0.001	0.44%	2.05%	Actinobacteria	Actinobacteria_acIV
River	Mainstem Dec.	3150	0.8401	0.001	0.30%	1.80%	Acidobacteria	
River	Mainstem Dec.	4527	0.8834	0.001	0.25%	1.58%	Betaproteobacteria	Betaproteobacteria_betVI
River	Mainstem Dec.	6766	0.5163	0.001	0.34%	1.24%	Verrucomicrobia	
River	Mainstem Dec.	2402	0.7233	0.001	0.19%	0.86%	Verrucomicrobia	
River	Mainstem Dec.	3282	0.7074	0.001	0.16%	0.75%	Acidobacteria	
River	Mainstem Dec.	4252	0.7695	0.001	0.14%	0.75%	Betaproteobacteria	
River	Mainstem Dec.	7060	0.4000	0.007	0.33%	0.70%	Rhodospirillales	
River	Mainstem Dec.	11015	1.0000	0.001	0.08%	0.64%	Rhizobiales	
River	Mainstem Dec.	10266	0.4942	0.002	0.21%	0.62%	Other Bacteria	Actinobacteria_acIV
River	Mainstem Dec.	7476	0.7034	0.001	0.13%	0.60%	Actinobacteria	
River	Mainstem Dec.	12568	0.4494	0.010	0.23%	0.56%	Verrucomicrobia	
River	Mainstem Dec.	11212	0.6622	0.001	0.10%	0.53%	Flavobacteria	
River	Tributaries May	8629	0.4019	0.001	1.43%	2.76%	Actinobacteria	Actinobacteria_acI
River	Tributaries May	2555	0.3934	0.001	0.81%	1.38%	Actinobacteria	Actinobacteria_acIV
River	Tributaries May	3685	0.4684	0.002	0.52%	1.21%	Betaproteobacteria	Betaproteobacteria_betII
River	Tributaries May	6842	0.3877	0.001	0.61%	1.13%	Actinobacteria	Actinobacteria_acI
River	Tributaries May	6128	0.4068	0.001	0.58%	1.13%	Actinobacteria	Actinobacteria_acIV
River	Tributaries May	10912	0.4871	0.002	0.45%	1.07%	Sphingobacteria	Bacteroidetes_bacI
River	Tributaries May	2258	0.4593	0.001	0.37%	0.78%	Verrucomicrobia	
River	Tributaries May	10731	0.4209	0.005	0.34%	0.75%	Actinobacteria	Actinobacteria_acIV
River	Tributaries May	3389	0.6392	0.001	0.17%	0.59%	Verrucomicrobia	Verrucomicrobia_verI-A
River	Tributaries May	3337	0.6150	0.001	0.21%	0.57%	Sphingobacteria	Bacteroidetes_bacI
River	Tributaries Sept. & Dec.	7260	0.3815	0.009	2.96%	6.51%	Synechococcus	
River	Tributaries Sept. & Dec.	9534	0.4019	0.001	2.22%	4.45%	Actinobacteria	Actinobacteria_acI
River	Tributaries Sept. & Dec.	8172	0.4734	0.001	1.67%	3.74%	Actinobacteria	Actinobacteria_acI
River	Tributaries Sept. & Dec.	3993	0.4957	0.001	1.23%	3.66%	Synechococcus	
River	Tributaries Sept. & Dec.	6557	0.3753	0.001	2.05%	3.53%	Actinobacteria	Actinobacteria_acI
River	Tributaries Sept. & Dec.	10037	0.5679	0.001	0.73%	2.21%	Sphingobacteria	Bacteroidetes_bacI
River	Tributaries Sept. & Dec.	10521	0.5678	0.001	0.44%	1.26%	Rhizobiales	Alphaproteobacteria_alfI
River	Tributaries Sept. & Dec.	4267	0.8244	0.001	0.22%	1.01%	Actinobacteria	Actinobacteria_acI
River	Tributaries Sept. & Dec.	3230	0.4485	0.003	0.34%	0.90%	Gammaproteobacteria	
River	Tributaries Sept. & Dec.	11206	0.7471	0.001	0.15%	0.76%	Gammaproteobacteria	
River	Tributaries Sept. & Dec.	151	0.4975	0.001	0.30%	0.74%	Actinobacteria	Actinobacteria_acI
River	Tributaries Sept. & Dec.	3333	0.4659	0.001	0.29%	0.71%	Rhizobiales	Alphaproteobacteria_alfI
River	Tributaries Sept. & Dec.	10449	0.7274	0.001	0.17%	0.69%	Actinobacteria	Actinobacteria_acIV
River	Tributaries Sept. & Dec.	7040	0.5232	0.001	0.12%	0.69%	Gammaproteobacteria	
River	Tributaries Sept. & Dec.	1965	0.5375	0.004	0.16%	0.68%	Sphingobacteria	Bacteroidetes_bacI
River	Tributaries Sept. & Dec.	7129	0.5698	0.001	0.22%	0.66%	Other Bacteria	
River	Tributaries Sept. & Dec.	12080	0.6446	0.002	0.11%	0.63%	Verrucomicrobia	
River	Tributaries Sept. & Dec.	8978	0.4519	0.001	0.23%	0.56%	Sphingobacteria	
River	Tributaries Sept. & Dec.	1073	0.9358	0.001	0.10%	0.55%	Other Bacteria	
River	Tributaries Sept. & Dec.	9568	0.6757	0.001	0.15%	0.53%	Sphingobacteria	Bacteroidetes_bacI
River	Tributaries Sept. & Dec.	10643	0.6372	0.001	0.12%	0.52%	Betaproteobacteria	Betaproteobacteria_betI
Plume whole & <0.2um	Low Salinity Plume	3993	0.6608	0.001	4.35%	13.64%	Synechococcus	
Plume whole & <0.2um	Low Salinity Plume	4502	0.4305	0.008	3.62%	6.66%	Gammaproteobacteria	
Plume whole & <0.2um	Low Salinity Plume	5886	0.7924	0.001	0.96%	4.03%	Flavobacteria	
Plume whole & <0.2um	Low Salinity Plume	9597	0.8563	0.001	0.80%	3.39%	Flavobacteria	
Plume whole & <0.2um	Low Salinity Plume	11451	0.4857	0.001	1.80%	3.38%	SAR11	
Plume whole & <0.2um	Low Salinity Plume	3990	0.4199	0.003	2.06%	3.14%	SAR11	
Plume whole & <0.2um	Low Salinity Plume	11178	0.4822	0.004	0.67%	2.69%	Gammaproteobacteria	
Plume whole & <0.2um	Low Salinity Plume	9285	0.5569	0.001	0.94%	2.48%	Gammaproteobacteria	
Plume whole & <0.2um	Low Salinity Plume	4960	0.7223	0.001	0.60%	1.99%	Rhodobacterales	

Plume whole & <0.2um	Low Salinity Plume	37	0.5723	0.001	0.46%	1.88%	Firmicutes
Plume whole & <0.2um	Low Salinity Plume	8885	0.7652	0.001	0.38%	1.70%	Sphingobacteria
Plume whole & <0.2um	Low Salinity Plume	8689	0.4288	0.004	0.86%	1.67%	SAR11
Plume whole & <0.2um	Low Salinity Plume	11116	0.7299	0.001	0.46%	1.66%	Flavobacteria
Plume whole & <0.2um	Low Salinity Plume	5309	0.8406	0.001	0.41%	1.65%	Flavobacteria
Plume whole & <0.2um	Low Salinity Plume	8316	0.6234	0.001	0.35%	1.33%	Rhodobacterales
Plume whole & <0.2um	Low Salinity Plume	6003	0.6069	0.001	0.26%	1.19%	Sphingobacteria
Plume whole & <0.2um	Low Salinity Plume	7946	0.5952	0.001	0.50%	1.16%	Gammaproteobacteria
Plume whole & <0.2um	Low Salinity Plume	4224	0.5504	0.001	0.47%	1.03%	Actinobacteria
Plume whole & <0.2um	Low Salinity Plume	11267	0.5344	0.002	0.30%	0.90%	Rhodobacterales
Plume whole & <0.2um	Low Salinity Plume	5406	0.7234	0.001	0.20%	0.68%	Actinobacteria
Plume whole & <0.2um	Low Salinity Plume	8919	0.5972	0.001	0.25%	0.66%	Sphingobacteria
Plume whole & <0.2um	Low Salinity Plume	8052	0.4390	0.002	0.15%	0.63%	Verrucomicrobia
Plume whole & <0.2um	Low Salinity Plume	4958	0.4998	0.001	0.14%	0.61%	Flavobacteria
Plume whole & <0.2um	Low Salinity Plume	5596	0.6848	0.001	0.13%	0.56%	Other Bacteria
Plume whole & <0.2um	Low Salinity Plume	10549	0.9254	0.001	0.12%	0.56%	Rhodobacterales
Plume whole & <0.2um	Low Salinity Plume	10994	0.5248	0.001	0.25%	0.55%	Sphingobacteria
Plume whole & <0.2um	DDA	11521	0.9960	0.001	0.76%	7.47%	Verrucomicrobia
Plume whole & <0.2um	DDA	1945	0.4356	0.003	0.68%	5.98%	SAR406
Plume whole & <0.2um	DDA	12446	0.8056	0.001	0.45%	3.20%	Flavobacteria
Plume whole & <0.2um	DDA	1011	0.3732	0.008	0.88%	3.16%	Deltaproteobacteria
Plume whole & <0.2um	DDA	10246	0.6704	0.001	0.67%	2.40%	Rickettsiales
Plume whole & <0.2um	DDA	2463	0.5059	0.001	0.66%	2.14%	Gammaproteobacteria
Plume whole & <0.2um	DDA	11690	0.4362	0.001	0.96%	2.04%	Rhodobacterales
Plume whole & <0.2um	DDA	5507	0.9982	0.001	0.18%	1.83%	Rhodobacterales
Plume whole & <0.2um	DDA	3266	0.8796	0.001	0.21%	1.40%	Verrucomicrobia
Plume whole & <0.2um	DDA	6515	0.0000	0.001	0.13%	1.30%	Verrucomicrobia
Plume whole & <0.2um	DDA	12243	0.7732	0.001	0.12%	1.20%	Verrucomicrobia
Plume whole & <0.2um	DDA	3806	0.0000	0.001	0.11%	1.11%	Flavobacteria
Plume whole & <0.2um	DDA	5528	0.8889	0.001	0.11%	1.11%	Verrucomicrobia
Plume whole & <0.2um	DDA	1012	0.6180	0.001	0.26%	1.04%	Synechococcus
Plume whole & <0.2um	DDA	4479	0.9934	0.001	0.10%	0.99%	Flavobacteria
Plume whole & <0.2um	DDA	12904	0.4438	0.003	0.35%	0.93%	Flavobacteria
Plume whole & <0.2um	DDA	494	0.5479	0.001	0.40%	0.87%	Rhodobacterales
Plume whole & <0.2um	DDA	9234	0.6377	0.001	0.24%	0.78%	Gammaproteobacteria
Plume whole & <0.2um	DDA	10959	0.3277	0.003	0.08%	0.72%	Calothrix
Plume whole & <0.2um	DDA	765	0.3982	0.005	0.09%	0.62%	Gammaproteobacteria
Plume whole & <0.2um	High Salinity Plume	9598	0.4463	0.002	8.96%	18.30%	Synechococcus
Plume whole & <0.2um	High Salinity Plume	1009	0.4089	0.001	0.88%	1.28%	Actinobacteria
Plume whole & <0.2um	High Salinity Plume	11419	0.5625	0.001	0.26%	0.72%	Flavobacteria
Plume whole & <0.2um	Plume Edge	6213	0.7672	0.001	13.68%	28.79%	Prochlorococcus
Plume whole & <0.2um	Plume Edge	12166	0.3650	0.001	12.23%	15.51%	SAR11
Plume whole & <0.2um	Plume Edge	6458	0.3621	0.002	2.87%	3.86%	SAR11
Plume whole & <0.2um	Plume Edge	1635	0.6284	0.001	1.71%	3.14%	SAR11
Plume whole & <0.2um	Plume Edge	12464	0.5598	0.001	0.91%	1.69%	SAR11
Plume whole & <0.2um	Plume Edge	6457	0.5281	0.001	0.39%	1.24%	Other Bacteria
Plume whole & <0.2um	Plume Edge	6211	0.5503	0.001	0.42%	1.13%	Actinobacteria
Plume whole & <0.2um	Plume Edge	3457	0.5824	0.001	0.21%	0.79%	Rickettsiales
Plume whole & <0.2um	Plume Edge	6910	0.3872	0.007	0.12%	0.79%	Other Bacteria
Plume whole & <0.2um	Plume Edge	10383	0.4177	0.004	0.44%	0.68%	Rickettsiales
Plume whole & <0.2um	Plume Edge	6856	0.4061	0.003	0.45%	0.66%	Rickettsiales
Plume whole & <0.2um	Plume Edge	7841	0.4287	0.001	0.48%	0.65%	Flavobacteria
Plume whole & <0.2um	Plume Edge	941	0.4473	0.002	0.39%	0.63%	Gammaproteobacteria
Plume whole & <0.2um	Plume Edge	9464	0.3885	0.007	0.42%	0.62%	SAR11
Plume whole & <0.2um	Plume Edge	9233	0.5147	0.001	0.18%	0.56%	Rhodospirillales
Plume >2.0um	Low Salinity Plume >2.0um	3993	0.9642	0.001	7.31%	36.05%	Synechococcus
Plume >2.0um	Low Salinity Plume >2.0um	5598	0.8002	0.002	1.40%	5.41%	Gammaproteobacteria
Plume >2.0um	Low Salinity Plume >2.0um	3990	0.8462	0.001	1.22%	5.07%	SAR11
Plume >2.0um	Low Salinity Plume >2.0um	5886	0.9170	0.002	0.75%	3.38%	Flavobacteria
Plume >2.0um	Low Salinity Plume >2.0um	1438	0.8955	0.001	0.61%	2.71%	Flavobacteria
Plume >2.0um	Low Salinity Plume >2.0um	11451	0.8446	0.001	0.65%	2.62%	SAR11
Plume >2.0um	Low Salinity Plume >2.0um	9597	0.9603	0.001	0.48%	2.34%	Flavobacteria
Plume >2.0um	Low Salinity Plume >2.0um	5406	0.9527	0.001	0.40%	1.94%	Actinobacteria
Plume >2.0um	Low Salinity Plume >2.0um	5596	0.9904	0.001	0.32%	1.66%	Alphaproteobacteria
Plume >2.0um	Low Salinity Plume >2.0um	7946	0.7050	0.001	0.53%	1.66%	Gammaproteobacteria
Plume >2.0um	Low Salinity Plume >2.0um	4958	0.7500	0.008	0.24%	1.24%	Flavobacteria
Plume >2.0um	Low Salinity Plume >2.0um	4224	0.6202	0.001	0.43%	1.10%	Actinobacteria
Plume >2.0um	Low Salinity Plume >2.0um	5410	1.0000	0.001	0.21%	1.10%	Flavobacteria
Plume >2.0um	Low Salinity Plume >2.0um	4960	0.8275	0.002	0.25%	0.96%	Rhodobacterales
Plume >2.0um	Low Salinity Plume >2.0um	9285	0.8652	0.001	0.23%	0.93%	Gammaproteobacteria
Plume >2.0um	Low Salinity Plume >2.0um	8689	0.5989	0.001	0.35%	0.90%	SAR11
Plume >2.0um	Low Salinity Plume >2.0um	6312	1.0000	0.001	0.17%	0.87%	Deferribacteres
Plume >2.0um	Low Salinity Plume >2.0um	6164	0.6309	0.001	0.19%	0.54%	Alphaproteobacteria
Plume >2.0um	DDA >2.0um	10959	0.8903	0.002	5.50%	24.97%	Calothrix
Plume >2.0um	DDA >2.0um	4479	0.9072	0.002	1.05%	4.85%	Flavobacteria
Plume >2.0um	DDA >2.0um	5507	0.9091	0.001	0.97%	4.51%	Rhodobacterales
Plume >2.0um	DDA >2.0um	11521	0.9138	0.001	0.86%	4.03%	Verrucomicrobia
Plume >2.0um	DDA >2.0um	8684	0.9454	0.002	0.56%	2.73%	Sphingobacteria
Plume >2.0um	DDA >2.0um	12446	0.9295	0.001	0.57%	2.68%	Flavobacteria
Plume >2.0um	DDA >2.0um	6162	0.6597	0.001	0.83%	2.65%	Flavobacteria
Plume >2.0um	DDA >2.0um	11345	0.8656	0.002	0.45%	1.89%	Deltaproteobacteria
Plume >2.0um	DDA >2.0um	12572	0.9149	0.001	0.26%	1.21%	Bacteroidetes
Plume >2.0um	DDA >2.0um	12768	0.7500	0.008	0.19%	1.01%	Deltaproteobacteria
Plume >2.0um	DDA >2.0um	9375	0.8889	0.002	0.21%	0.90%	Cyanobacteria
Plume >2.0um	DDA >2.0um	5002	0.8883	0.003	0.19%	0.85%	Rickettsiales
Plume >2.0um	DDA >2.0um	4501	0.9211	0.001	0.17%	0.79%	Rickettsiales

Plume >2.0um	DDA >2.0um	2760	0.9420	0.001	0.15%	0.73%	Sphingobacteria
Plume >2.0um	DDA >2.0um	3806	1.0000	0.001	0.13%	0.68%	Flavobacteria
Plume >2.0um	DDA >2.0um	765	0.7237	0.007	0.12%	0.62%	Gammaproteobacteria
Plume >2.0um	DDA >2.0um	5528	1.0000	0.001	0.12%	0.62%	Verrucomicrobia
Plume >2.0um	Deep >2.0um	648	0.5902	0.007	0.87%	3.95%	Gammaproteobacteria
Plume >2.0um	Deep >2.0um	12622	0.6943	0.008	0.50%	2.93%	Rhodobacterales
Plume >2.0um	Deep >2.0um	3846	0.5695	0.009	0.58%	2.71%	Gammaproteobacteria
Plume >2.0um	Deep >2.0um	9989	0.6276	0.009	0.12%	0.56%	Actinobacteria
Plume >2.0um	High Salinity Plume >2.0um	5093	0.9759	0.001	1.79%	5.25%	Sphingobacteria
Plume >2.0um	High Salinity Plume >2.0um	4407	0.8160	0.001	1.10%	2.85%	Sphingobacteria
Plume >2.0um	High Salinity Plume >2.0um	37	0.8571	0.002	0.59%	1.77%	Firmicutes
Plume >2.0um	High Salinity Plume >2.0um	10994	0.4963	0.001	0.62%	1.13%	Sphingobacteria
Plume >2.0um	High Salinity Plume >2.0um	12049	0.8571	0.002	0.34%	1.03%	Flavobacteria
Plume >2.0um	High Salinity Plume >2.0um	3896	0.7590	0.002	0.27%	0.74%	Verrucomicrobia
Plume >2.0um	High Salinity Plume >2.0um	8432	0.9414	0.001	0.25%	0.72%	Planctomycetes
Plume >2.0um	High Salinity Plume >2.0um	11116	0.7554	0.004	0.23%	0.63%	Flavobacteria
Plume >2.0um	Plume Edge >2.0um	7105	0.7423	0.001	4.65%	13.24%	Gammaproteobacteria
Plume >2.0um	Plume Edge >2.0um	6213	0.8754	0.001	1.27%	4.67%	Prochlorococcus
Plume >2.0um	Plume Edge >2.0um	4743	0.7679	0.008	0.62%	1.76%	Rhodobacterales
Plume >2.0um	Plume Edge >2.0um	5088	0.8356	0.003	0.50%	1.56%	Alphaproteobacteria
Plume >2.0um	Plume Edge >2.0um	7841	0.6731	0.002	0.50%	1.29%	Flavobacteria
Plume >2.0um	Plume Edge >2.0um	11865	0.7000	0.007	0.33%	0.90%	Planctomycetes
Plume >2.0um	Plume Edge >2.0um	4744	0.8000	0.001	0.18%	0.77%	Gammaproteobacteria
Plume >2.0um	Plume Edge >2.0um	2071	0.7234	0.008	0.18%	0.61%	Flavobacteria

Supplemental Figure S1. Average Alpha diversity (Catchall) of Amazon River and plume bacterial communities. Error bars indicate standard error.

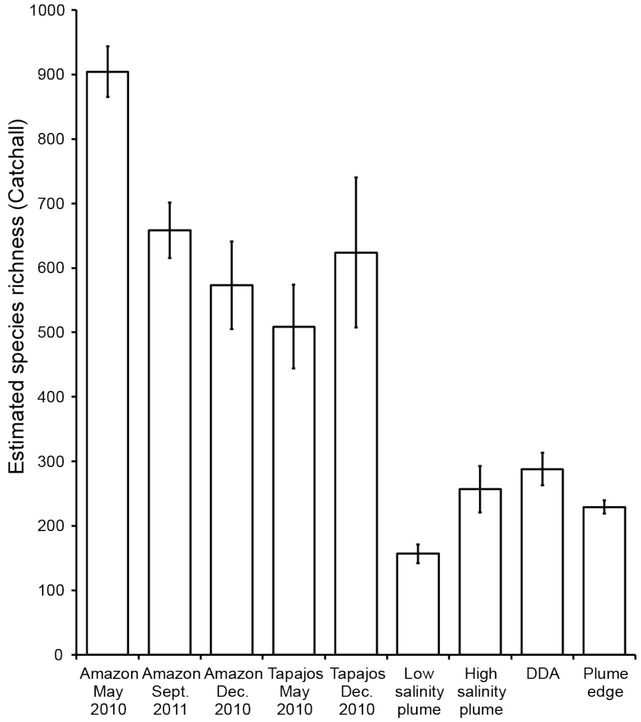

Supplemental Figure S2. Average taxonomic diversity of bacterial communities for each major environmental grouping in the Amazon river and plume.

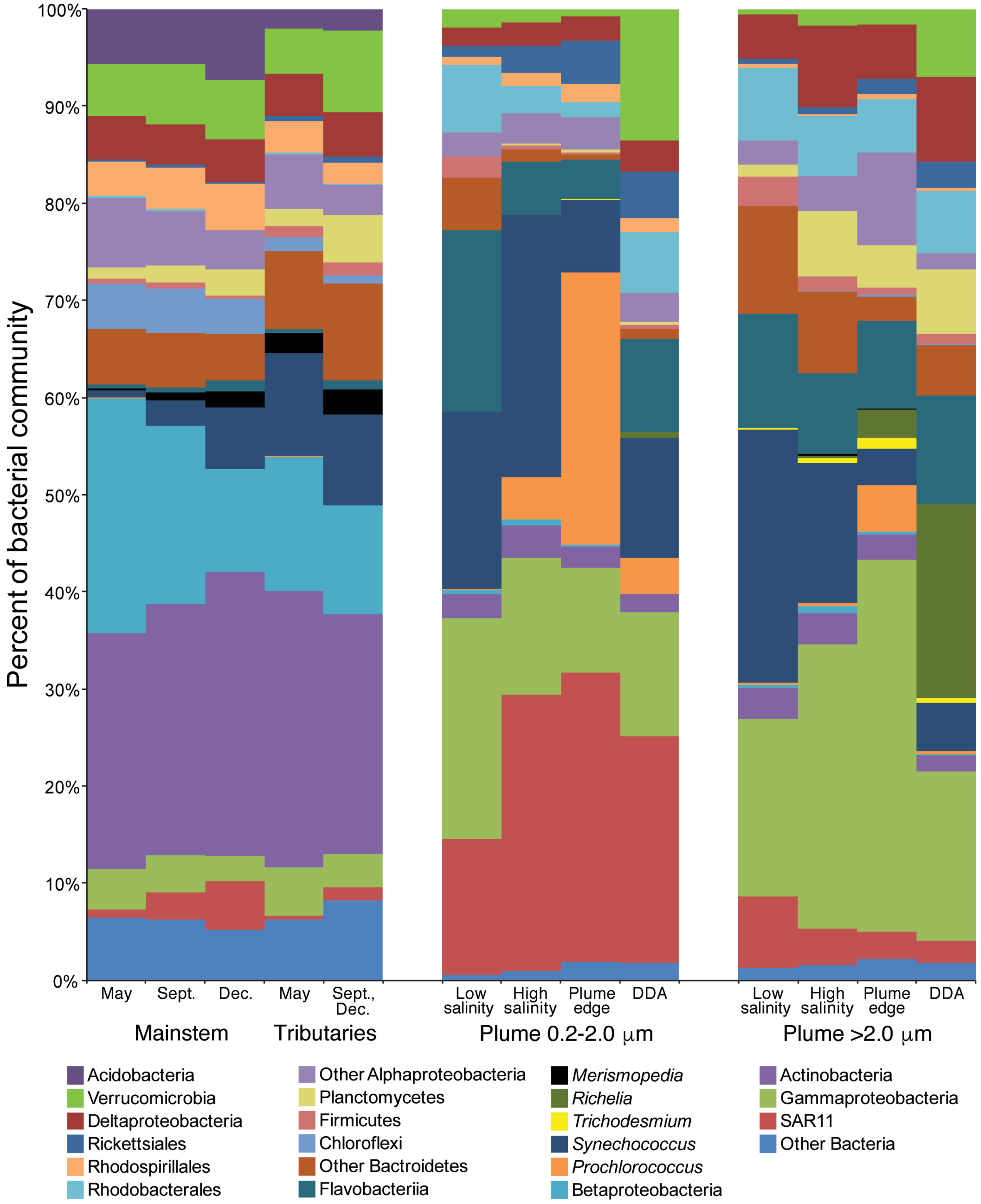

Supplemental Figure S3. Bubble plot of river indicator taxa identified for the five sample groups plotted against indicator value (IV) and sized to show the average relative abundance of each taxa in that set of samples. Plot shows all identified indicator taxa with IV>0.3, *p*<0.01, and relative abundance > 0.001 within the indicated sample group.

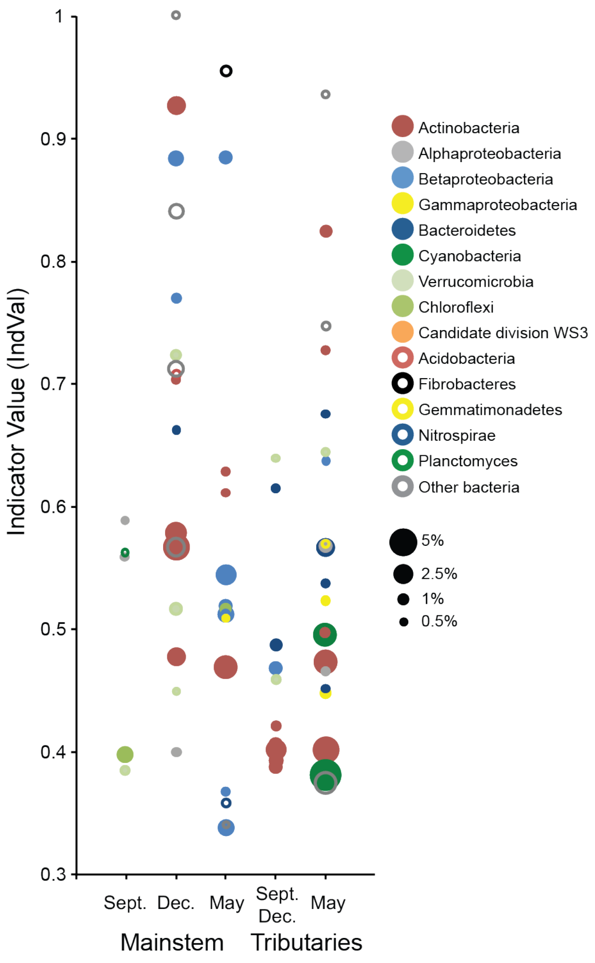

Supplemental Figure S4. Bubble plot of indicator taxa for plume (A) whole water and 0.2-2.0 µm size fractions, and (B) >2.0 µm size fraction identified for the five sample groups plotted against indicator value (IV) and sized to show the average relative abundance of each taxa in that set of samples. Plot shows all identified indicator taxa with IV>0.3, p<0.01, and relative abundance > 0.001 within the indicated sample group.

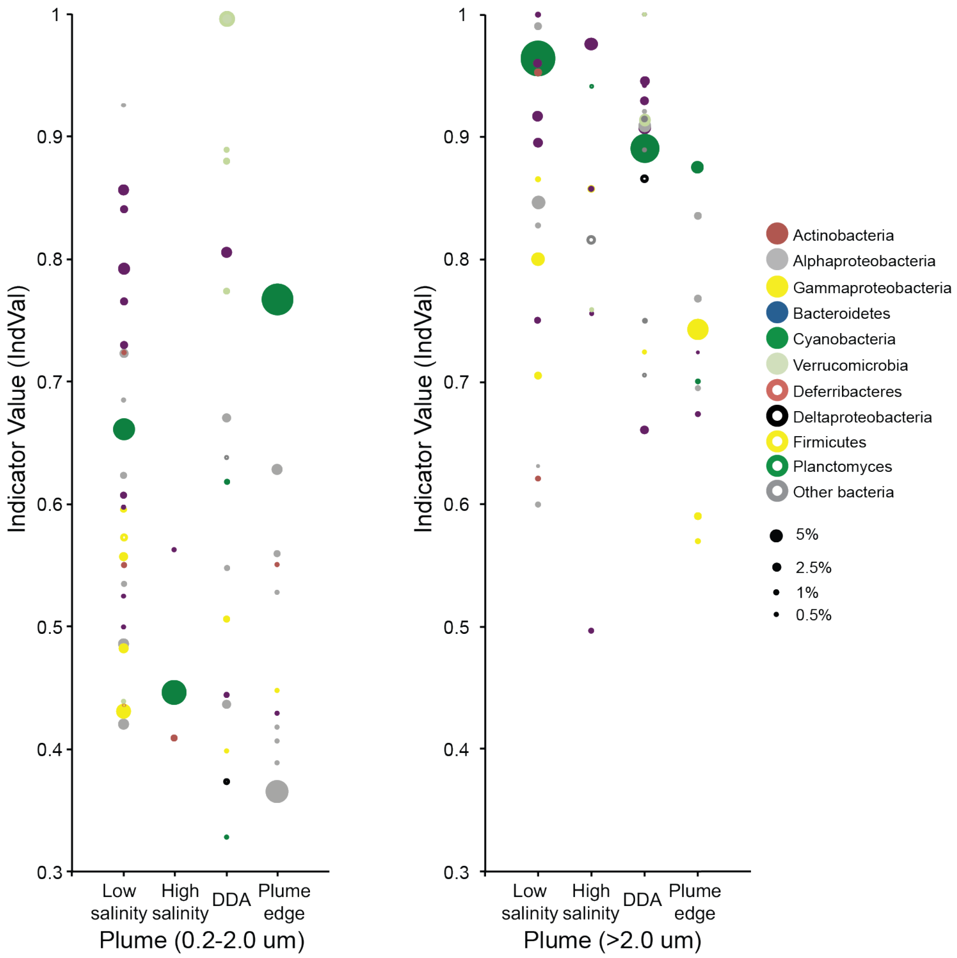

Supplemental Figure S5. Multidimensional scaling diagrams of Amazon Plume and River bacterial community composition betadiversity calculated as Unweighted and Weighted UniFrac distances. Closed symbols and '*' represent unfractionated samples and free-living bacteria (0.2-2.0 µm). Open symbols and 'X' represent particle-attached bacteria (>2.0 µm).

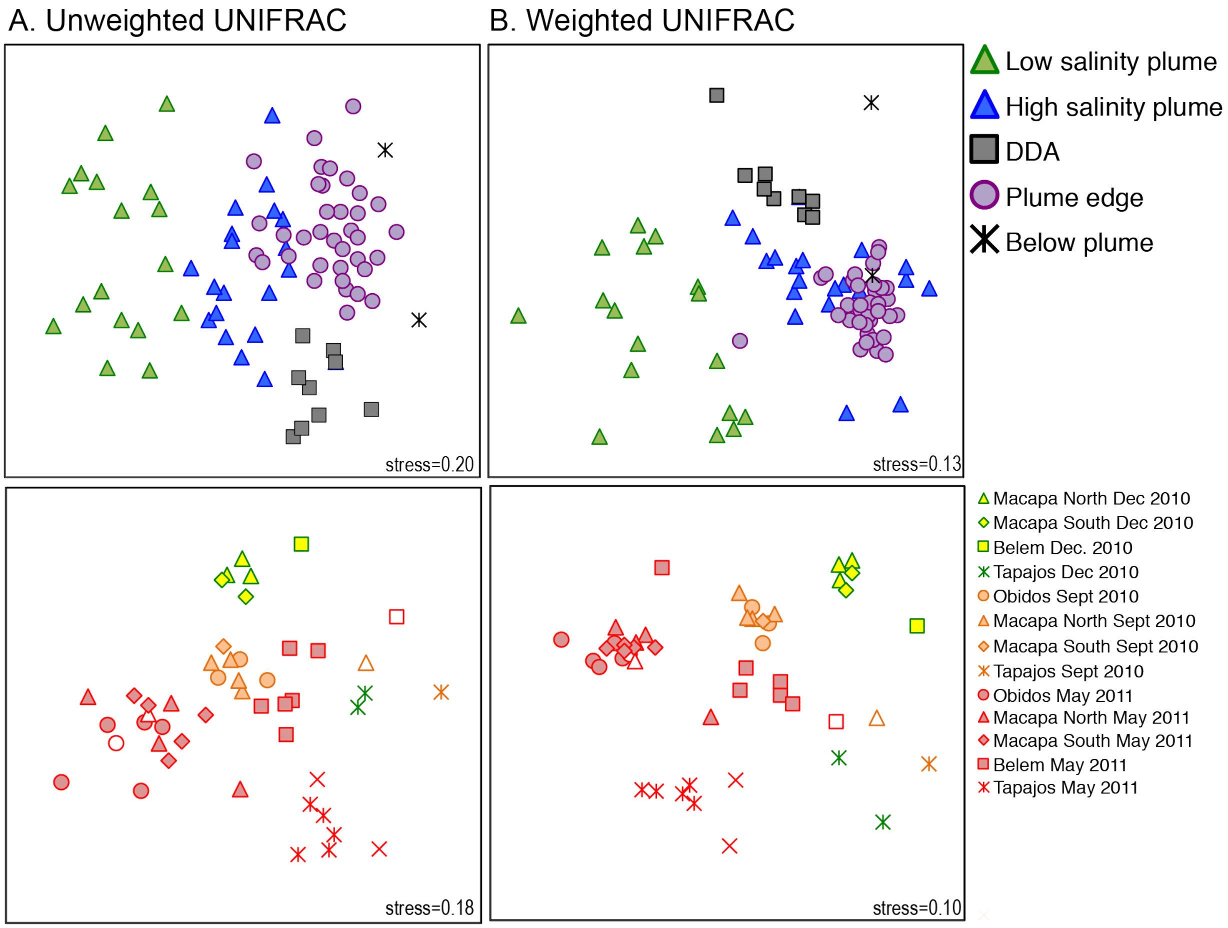

Supplemental Figure S6. Multidimensional Scaling (MDS) diagrams showing Unweighted UniFrac (A) and Weighted UniFrac (B) similarity among bacterial communities in all samples collected for this study, communities from the Columbia River, estuary, plume, and surface ocean (Fortunato et al. 2013), communities from six arctic rivers (Crump et al. 2009) and from the Bering Strait.

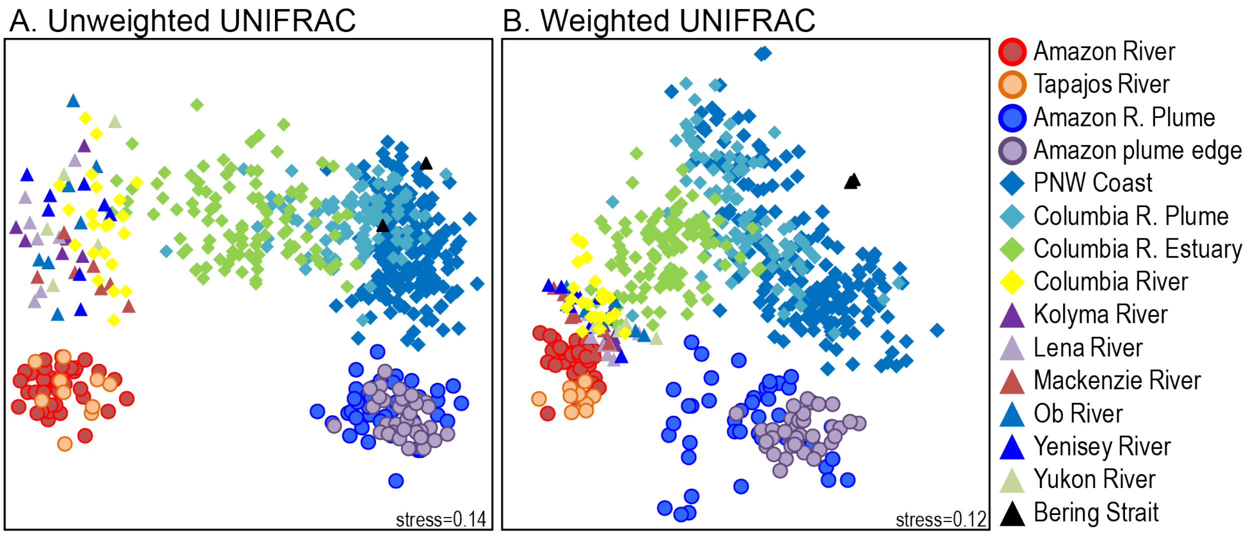

Supplement: Supplementary file 1 [file Presentation_1.pdf]
